# The global prevalence of depression, suicide ideation, and attempts in the military forces: a systematic review and Meta-analysis of cross sectional studies

**DOI:** 10.1186/s12888-021-03526-2

**Published:** 2021-10-15

**Authors:** Yousef Moradi, Behnaz Dowran, Mojtaba Sepandi

**Affiliations:** 1 Health Research Center, Life Style Institute, Baqiyatallah University of Medical Sciences, Tehran, Iran; 2 Social Determinants of Health Research Center, Research Institute for Health Development, Kurdistan University of Medical Sciences, Sanandaj, Iran; 3 Behavioral Sciences Research Center, Life style institute, Baqiyatallah University of Medical Sciences, Tehran, Iran

**Keywords:** Suicide ideation, Suicide attempts, Depression, Military, Systematic review and Meta-analysis

## Abstract

**Background:**

Given the wide range of depressive disorders, suicidal ideation and suicide attempts in various military studies around the world, determining the exact prevalence of these disorders in line with health planning as well as care and treatment service designing for military forces can be useful. The aim of the present meta-analysis was to determine the pooled prevalence of depressive disorders, suicide thoughts, and attempts in the military.

**Methods:**

The present systematic review and meta-analysis study was performed based on PRISMA criteria in 5 steps of the search strategy, screening and selection of articles, data extraction, evaluation of article quality and meta-analysis. International databases (PubMed (Medline), Scopus, Web of science, Embase (Elsevier), PsycInfo (Ovid), Cochrane CENTRAL (Ovid)) were searched using related keywords extracted from Mesh and Emtree. After screening and final selection of articles, data were extracted and qualitative evaluation was performed using the NOS checklist.

**Results:**

The results of meta-analysis showed that the prevalence of depression in active military forces and veterans was 23% (%95 CI: 20–26%) and 20% (%95 CI: 18–22%), respectively. In addition, the prevalence of suicidal ideation and attempts in the military was 11% (%95 CI: 10–13%) and 11% (%95 CI: 9–13%), respectively. The prevalence of suicide ideation and attempts in drug-using military was 18% (%95 CI: 7–33%) and 30% (%95 CI: 23–36%), respectively. The prevalence of suicidal ideation and attempts in military consuming alcohol were 9% (%95 CI: 4–13%) and 8% (%95 CI: 7–10%), respectively. In militaries with AIDS / HIV, the prevalence of suicide attempts was 5% (%95 CI: 4–8%).

**Conclusion:**

Therefore, it is necessary to develop and design training and intervention programs in order to increase the awareness of the military, especially veterans, to prevent the occurrence of suicide and depression.

**Supplementary Information:**

The online version contains supplementary material available at 10.1186/s12888-021-03526-2.

## Background

Mental health is one of the basic pillars of health that requires a useful, effective and satisfactory individual life [1]. Promoting the mental health of a society requires the dynamism and growth of that society [2]. Paying attention to mental health in all areas of life, including personal, social and professional ones, is important and debatable. One of the areas in which mental health is concerned is the job and profession. Based on the available findings, mental disorders are one of the most important and significant causes of diseases and it was predicted that in 2020 the share of mental and neurological disorders in the total burden of diseases would increase by 50% [3–5]. Therefore, attention to mental health is important in all areas of the individual, social and professional life [6, 7]. One of the important stressful environmental stimuli that can cause chronic stress and significantly affect people’s psyche is the type of the job in which a person is engaged so that if the stress caused by the work environment becomes excessive, it can cause physical and psychological effects on the individual and his/her family. It can be said that it endangers the health of the individual and threatens the organizational goals and leads to a decrease in the quality of the individual’s performance. Research has shown that several factors affect job stress [8–10]. These include shift work, or jobs which are full of environmental stress. If a person is not able to cope with the stressors of his/her job, he/she will suffer from multiple physical, psychological and behavioral consequences. In this regard, the military forces of different countries perform different missions according to the conditions of the region and their countries, but during this decade, in order to provide higher defense capability and presence at greater depths and distances away from the origin, military forces need to design and make tools with higher ranges and quality, which need their own engineering and ergonomic requirements [11–13]. One of the most important issues in this field, which can be the first question and has caused intellectual and executive concern of military officials and commanders, is to identify and implement methods to increase the durability and maintain the performance of military personnel so that during increasing mission time, their efficiency will not be disrupted or effectively reduced [14, 15]. This is where the role of military psychology and psychological variables affecting the effectiveness of military forces become clearer [16, 17]. Psychological assessment and mental disorders are very important among military personnel because war, living in operational conditions, multiple combat missions, being away from the family, captivity, wounding and environmental restrictions, as well as cultural differences are always parts of the military life. Therefore, due to this type of lifestyle, burnout, job stress and various mental disorders such as depression and suicide are very common among them [18, 19]. For this reason, conducting epidemiological and psychological research among military personnel is of great importance. In addition, accurately determining the prevalence of mental disorders in this group can help health policy makers and health professionals to take more effective and appropriate control and treatment measures [20, 21]. On the other hand, the military forces’ awareness of the occurrence of these disorders can be effective in performing appropriate health behaviors, suitable lifestyle changes, and ultimately in preventing further occurrence of these disorders. So far, various descriptive and analytical studies have been conducted in the world with the aim of determining the prevalence of mental disorders, especially depression and suicide in servicemen in various fields such as naval, land and air forces, but the results of these studies were very contradictory. So far, various studies with different sample sizes in the world have been conducted to determine the prevalence of depression and suicide (thoughts or attempted) in the military, but the results of these studies showed the wide prevalence of these consequences in the military and so far, the exact prevalence of them in these communities has not been determined [7, 22–24]. The unavailability of the exact prevalence of depression and suicide in the military prevents the development of appropriate mental health programs and interventions for the military. On the other hand, the burden of these diseases and mental illnesses in the military is still questionable due to the unavailability of an accurate prevalence [25–27]. Accurately determining the prevalence of depression and suicide in the military can help determine the burden of mental illnesses in the military, plan mental health, develop and implement mental health interventions, as well as allocate health resources. Also, it makes health policy makers and the health sector aware of the level of mental illnesses in the military. In this study, the authors aimed to accurately estimate the prevalence of depression, suicide thoughts and attempts in the world’s military.

## Methods

This systematic review and meta-analysis was based on the standards Preferred Reporting Items for Systematic Reviews and Meta-Analyzes (PRISMA) and Meta-analyzes of Observational Studies in Epidemiology (MOOSE) [28–30]. The protocol of this study had been registered in the International Prospective Register of Systematic Reviews (PROSPERO), under the registration number of CRD42021233973.

### Search syntax and search strategy

This study was a systematic review and meta-analysis that aimed to accurately determine the prevalence of depression, suicide thoughts, and suicide attempts in the military. Finding of articles published from January 1990 to December 2020 was done in 5 electronic databases (PubMed (Medline), Scopus, Web of science, Embase (Elsevier), PsycInfo (Ovid), Cochrane CENTRAL (Ovid)) using the main keywords of Depression (synonymous with “Depressively”, “Depressive Disorder”, “Depressed”, “Depressive Symptoms”, “Emotional Depression”, “Unipolar Depression”, “Neurotic Depression”, “Depressive Syndromes”, “Endogenous Depression”, and “Depressive Neurosis)”, suicide thoughts and attempts (with synonyms of “Suicide”, “Suicidality”, “Attempted Suicide”, “Para Suicide”, “Completed Suicide”, and “Thoughts of Suicide”), as well as Military people (with synonyms of “Armed Forces Personnel”, “Military Personnel”, “Air Forces Personnel”, “Veterans”, “Submariners”, “Marines”, “Navy Personnel”, “Sailors”, “Soldiers”, “Military Deployment”, and “Coast Guard “) (Supplement File). Gray Literature-related sites and databases such as Google Scholar, World Health Organization (WHO) were also searched. The search was generally done in google scholar in the advanced section, then the first 10 pages of the results were reviewed and matched with the final selected articles so that any article was not lost. For the World Health Organization website, international or national reports, the references of which were reviewed, were generally searched on the main website using main keywords, i.e. depression and suicide, then the keyword of military was considered in the study. The manual search in this article was performed by checking the reference lists of the articles. In this way, the references of the selected articles were scanned very quickly so that a relevant article would not be missed. In this review articles with English language were included.

### Eligibility criteria’s

Inclusion criteria contained the following:

- Cross-sectional studies whose main purpose was to estimate and determine the prevalence (frequency or percentage) of depression and suicide (thoughts or attempts) in the military.
- Cross-sectional studies that measured depression and suicide (suicidal ideation or attempts) in the military using accredited tools.
- Cross-sectional studies in which the study population was military personnel (serving or retired). Individuals who had been employed by the Army, Air Force, and Navy or retired from any of these organizations. In addition, servicemen who had fought in foreign wars (such as the wars in Afghanistan, Syria, Iraq, and Vietnam) would be considered military forces (active or retired) if surveyed in the selected studies (then they were separately analyzed in subgroup analyzes).

In this review articles with English language were included.

Exclusion criteria contained cross-sectional studies that had reported the desired outcomes (depression and suicide) on a crude average with standard deviation. Their target population was not military and they had not provided a precise definition of the military. In addition, studies other than cross-sectional ones, such as cohort studies, case studies, retrospective, or prospective studies with the cohort base, clinical trials, systematic reviews, letters to the editor, editorial, and survey studies over 5 years were excluded from the research.

### Screening and selection of articles

A definition was not included in the inclusion criteria for measuring suicide (suicide attempts or suicidal ideation) and depression, so the authors decided to screen and select articles, then based on the various tools (like standard questionnaires, the DSM-IV criteria or clinical findings measuring) used in the selected studies to measure depression and suicide, to perform subgroup analysis whose results were presented in the analysis tables.

First, an Endnote library (Version 8) was created to collect articles, remove duplicates, and review titles and abstracts. In the first screening step, the review of titles and abstracts was independently done by one of the researchers (YM) and 10% of the reviewed articles were randomly reviewed by the second researcher (MS) and the differences were resolved by discussing and referring to the third person (BD) if necessary. The screened references were selected for full-text review if they contained the desired information in their title or abstract. In the next step, the full text was separately reviewed by two of the authors. Data were extracted from the eligible studies and entered into Excel 2016.

### Data extraction

In order to extract the data, first a checklist was prepared with the opinion of experts in relation to the data extracted from the articles and then the data were extracted. Required information included author’s name, year of publication of articles, statistical population of study, country of study, type of study, instrument for measuring depression and suicide disorders in the military, sample size, average age of military personnel and quality evaluation score of primary studies. The data extraction was independently developed and conducted by two of the authors (YM and MS).

### Quality assessment

Two of the authors (YM and MS) conducted a qualitative evaluation of the studies based on the Newcastle - Ottawa Quality Assessment Scale (NOS) checklist [31, 32]. This checklist has designed to evaluate the quality of observational studies, especially cross-sectional ones. This tool examined each study with 6 items in three groups, including: how to select study samples, how to compare and analyze study groups, and how to measure and analyze the desired outcome. Each of these items was given a score of 1 if it was observed in the studies, and the maximum score for each study was 9 points. In case of discrepancies in the score assigned to the published articles and for reaching an agreement, the discussion method and the third researcher (BD) were used.

### Statistical methods

The number of patients with the desired outcome (depression or suicide) was extracted from the total sample size in each of the studies to perform the *Metaprop* order. In this research, the model of DerSimonian-Liard random effects was used to estimate the pooled prevalence of depression and suicide (estimate of 95% confidence interval) in military personnel. Cochrane Q and I2 tests were used to investigate the heterogeneity and variance between the studies selected for meta-analysis. According to the Cochrane criteria and I2 index, the amount of heterogeneity was divided into 4 categories: 0 to 40% (might not be important), 30 to 60% (may represent moderate heterogeneity), 50 to 90% (may represent substantial heterogeneity), and finally 75% and above (considerable heterogeneity) [33–36]. The L’Abbé Plot diagram was used to investigate this heterogeneity. Subgroup analysis was also used to find the source of heterogeneity (gender, service status (active or veteran) and health status of the military as well as sampling types, outcome measurement tools and finally the country). The Funnel Plot diagram and Egger test were used to check and determine the publication bias. The interpretation of the Egger test is that if the *P* value is significant, it can be interpreted that the publication bias has occurred, otherwise no bias has occurred. In addition, the Funnel diagram was used to express this bias. All analyzes were performed in STATA software, version 16.

## Results

### Qualitative results

**Fig. 1:**
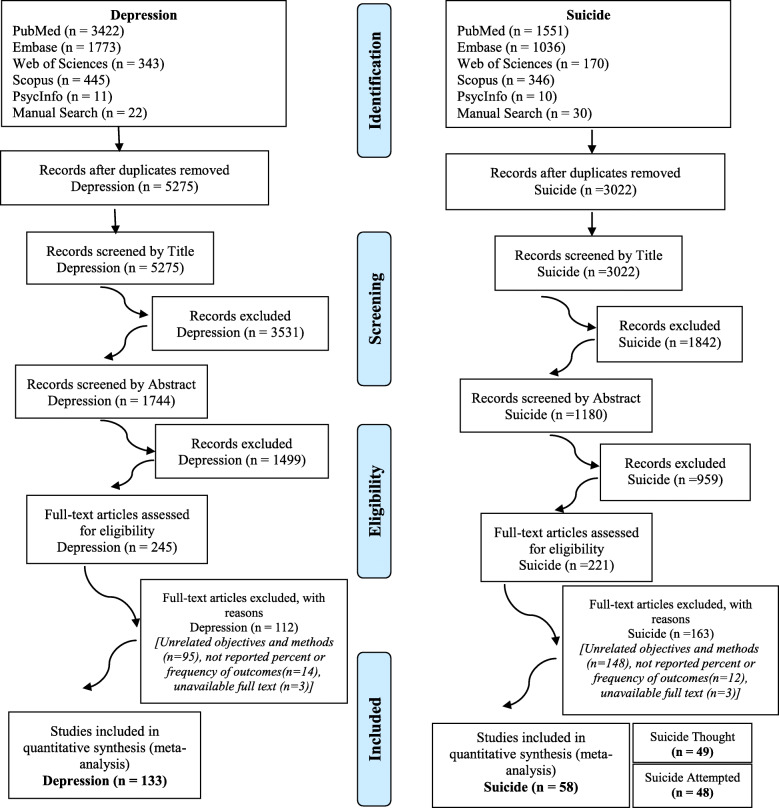
The Search Strategy Outputs and Screening Process based on Title, Abstract, and Full Text

After completing the search strategy, and eliminating duplicates in EndNote software, 5275 articles related to depression and 3022 articles related to suicide in the military of the world remained. After screening based on their titles and abstracts, 245 articles on depression and 221 articles on suicide remained in the study. Screening was performed based on the full texts of the articles, and finally 112 articles on depression and 163 articles on suicide were removed. Finally, 133 articles on depression and 58 articles on suicide in the military remained, which entered the meta-analysis. Of the suicide articles, 48 ​​were about suicide attempts and 49 were about suicidal ideation. Some of these articles reported both suicidal ideation and suicide attempts (Fig. 1). All characteristics extracted from selected studies were separately reported in Tables 1 and 2 based on the outcome of depression and suicide.

**Table 1:** The study characteristics of included studies about depression

Authors (Years)	Country	Type of Sampling (Type of Study)	Study Population	Depression Assessment Method	Age (Mean)	Sample size	Prevalence of Depression (%)	NOS Score
Tredgold, R. F. (1941)(65)	UK	Convenience Sampling (CS)	Army men	Clinical Symptoms (Interviews)	–	274	70 (25.54%)	6
Helzer, J. E. et al. (1976) (66)	USA	Random Sampling (CS)	Army men	Clinical Symptoms (Interviews)	–	470	122 (26%)	7
Levine, M. E. (1982) (67)	USA	Convenience Sampling (CS)	Army men	Beck Depression Inventory (BDI)	17	200	36 (18%)	6
Deeken, M. G. et al. (1987) (68)	USA	Convenience Sampling (CS)	Army men	Zung Self-Rating Depression Scale	–	298	47 (15.77%)	7
Ritchie, E. C. et al. (1992) (69)	USA	Random Sampling (CS)	Army men with HIV	Clinical Symptoms (Interviews) DSM-III-R	–	50	21 (42%)	7
Brown, G. R. et al. (1993) (70)	USA	Random Sampling (CS)	Air Forces men with HIV	Structured Interview Guide for the Hamilton Anxiety and Depression Scales (SIGH-AD)	35	442	99 (22.4%)	8
McCarroll, J. E. et al. (1993) (71)	USA	Convenience Sampling (CS)	Army men and women	Clinical Symptoms (Interviews)	25.4	1835	87 (4.7%)	8
						Male(1565)	59 (3.8%)	
						Female(270)	52 (19.3%)	
Perconte, S. T. et al. (1993) (72)	Russia	Convenience Sampling (CS)	Army men and women	Beck Depression Inventory (BDI)	29.25	591	146 (24.70%)	7
Serfaty, E. et al. (1995) (73)	Argentina	Random Sampling (CS)	Army men and women	NR	NR	553	25 (4.5%)	7
Lish, J. D. et al. (1996) (74)	USA	Random Sampling (CS)	Army men and women	Brief self-report questionnaire (SCRENNER)	21.2	669	38 (5.81%)	7
Long, N. et al. (1996) (75)	New Zealand	Random Sampling (CS)	Army men	Beck Depression Inventory (BDI)	50	751	11 (1.46%)	7
Schwartz, D. A. et al. (1997) (76)	USA	Random Sampling (CS)	Non-Persian Gulf War (PGW) military personnel	Self-report	–	923	157 (17%)	6
Schwartz, D. A. et al. (1997) (76)	USA	Random Sampling (CS)	Persian Gulf War (PGW) military personnel	Self-report	–	923	99 (10.9%)	6
David, D. et al. (1999) (77)	Croatia	Convenience Sampling (CS)	Veterans after participation in Homeland War in Croatia	The Structured Clinical Interview Diagnostic and Statistical Manual (SCID)	36.2	91	35 (38.5%)	7
Hankin, C. S. et al. (1999) (78)	USA	Random Sampling (CS)	Men Veterans	Center for Epidemiologic Studies Depression Scale (CES-D Scale)	62	2160	676 (31.3%)	7
Hourani, L. L. et al. (1999) (79)	USA	Random Sampling (CS)	Men and Women in the Navy and Marine Corps	Center for Epidemiologic Studies Depression Scale (CES-D Scale)	20–64	782	125 (16.08%)	7
						Male (321)	29 (9%)	
						Female (452)	99 (22%)	
Curran, G. M. et al. (2000) (80)	USA	Random Sampling (CS)	Men Veterans	(Beck Depression Inventory)BDI(	43	298	116 (39%)	7
Menon, A. S. et al. (2000) (81)	USA	Convenience Sampling (CS)	Men Veterans	The Structured Clinical Interview for DSM-III-R (SCID-III-R)	55	295	59 (22.8%)	6
Kozaric-Kovacic, D. et al. (2001) (82)	Croatia	Random Sampling (CS)	Men Veterans	The Hamilton Depression Rating Scale (HAMD)	34	249	77 (31%)	7
Sayar, K. et al. (2001) (83)	Turkey	Random Sampling (CS)	Men Soldiers	(Beck Depression Inventory)BDI(	22.7	40	13 (32.5%)	7
Hunter, C. L.et al. (2002)(84)	USA	Random Sampling (CS)	Active Duty	The Patient Health Questionnaire (PHQ) (the self-report version of the PRIME-MD)	54.15	337	19 (5.6%)	7
Karel, M. J. et al. (2002)(85)	USA	Random Sampling (Survey Study)	Men Veterans	The Geriatric Depression Scale (GDS)- 15 item	69.7	967	236 (24.4%)	7
				Hamilton Depression Rating Scale (HDRS)-24 item	69.7	967	94 (9.7%)	
Kilbourne, A. M. et al. (2002)(86)	USA	Random Sampling (CS)	Veterans with HIV infection	The 10-item Centers for Epidemiologic Studies Depression Scale (CES-D)	49	881	405 (46%)	7
Lehman, C. L. et al. (2002)(87)	USA	Convenience Sampling (CS)	Veterans with Hepatitis C	The Beck Depression Inventory (BDI)	49	120	53 (44.2%)	6
Muir, A. J. et al. (2002)(88)	USA	Convenience Sampling (CS)	Veterans with Hepatitis C	The Center for Epidemiological Studies Depression (CES-D) scale	47.3	100	12 (12%)	6
Nguyen, H. A. et al. (2002)(89)	USA	Convenience Sampling (CS)	Veterans with Hepatitis C	Clinical Symptoms (Interviews)	46.5	118	73 (62%)	6
Black, D. W. et al. (2004)(90)	USA	Convenience Sampling (CS)	Veterans	Clinical Symptoms (Interviews) DSM-III-R	39.3	602	192 (32%)	6
Gerson, S. et al. (2004) (91)	USA	Convenience Sampling (CS)	Elderly veterans (Male)	Mental Health Inventory (MHI)	69.6	839	273 (32.5%)	8
Rowan, P. J. et al. (2004)(92)	USA	Convenience Sampling (CS)	Veterans with Hepatitis C	The Zung Self-report Depression Scale (SDS)	51	580	93 (16%)	7
Smith, T. C. et al. (2004)(93)	USA	Random Sampling (CS)	US Military	The PRIME-MD Patient Health Questionnaire (PHQ)	55	8893	1642 (18.5%)	8
Vafaee, B.et al. (2004)(94)	Iran	Convenience Sampling (CS)	Disabled veterans male	The Zung Self-report Depression Scale (SDS)	38	100	71 (71%)	5
Forman-Hoffman, V. L. et al. (2005) (95)	USA	Convenience Sampling (CS)	Veterans	Structured Clinical Interview for DSM Disorders (SCID-IV)	39.1	602	85 (14.11%)	6
Goulet, J. L. et al. (2005)(96)	USA	Convenience Sampling (Re)	Veterans with HIV	–	47.1	20,627	5776 (28%)	7
			Veterans with Hepatitis C	–	46.9	4489	1975 (44%)	
Rowan, P. J. et al. (2005)(97)	USA	Convenience Sampling (CS)	Veterans with Hepatitis C	The Beck Depression Inventory (BDI)	52	62	6 (10%)	5
Williams, R. M. et al. (2005)(98)	USA	Convenience Sampling (CS)	Veterans with Multiple sclerosis	The Beck Depression Inventory (BDI)	55.1	451	100 (22.2%)	7
Xiong, H. et al. (2005)(99)	China	Random Sampling (CS)	Young adult males during their 8 week field military training	The Zung Self-report Depression Scale (SDS)	20	1107	279 (25.2%)	6
Grieger, T. A. et al. (2006)(100)	USA	Convenience Sampling (CS)	U.S. soldiers were injured in combat	The nine-item Patient Health Questionnaire depression scale	26.94	301	28 (9.3%)	5
Hoge, C. W. et al. (2006)(101)	USA	Random Sampling (CS)	Army soldiers and Marines	The PRIME-MD Patient Health Questionnaire (PHQ)	31.2	303,905	15,930 (5.24%)	8
Kress, A. M. et al. (2006)(102)	USA	Random Sampling (CS)	U.S. Military personnel	Burnam Screen	–	4227	844 (20%)	8
Pflanz, S. E. et al. (2006)(103)	USA	Convenience Sampling (CS)	Military Personnel	Depression Checklist	28.7	780	141 (18%)	7
Dove, M. B. et al. (2007)(104)	USA	Convenience Sampling (CS)	Women Entering a Military Substance Use Disorder	Depression Checklist	–	86	67 (78%)	5
Kolkow, T. T. et al. (2007)(105)	USA	Convenience Sampling (CS)	Army soldiers	The PRIME-MD Patient Health Questionnaire (PHQ)	34.30	100	5 (5%)	5
Warner, C. M. et al. (2007)(106)	USA	Convenience Sampling (CS)	Military Personnel	The PRIME-MD Patient Health Questionnaire (PHQ)	20.9	1090	173 (15.9%)	6
			Male		20.9	955	143 (15%)	
			Female		21	135	30 (22.2%)	
Hoge, C. W. et al. (2008) (107)	USA	Convenience Sampling (CS)	Army individual	The PRIME-MD Patient Health Questionnaire (PHQ)	–	1885	275 (15%)	6
			Marine individual			775	114 (14.7%)	
Iversen, A. C. et al. (2009)(108)	UK	Random Sampling (CS)	UK military personnel in service at the time of the 2003 Iraq War	The PRIME-MD Patient Health Questionnaire (PHQ)	35	821	223 (27.2%)	8
Kline, A. et al. (2009)(109)	USA	Convenience Sampling (CS)	Vietnam veterans with Substance Use Disorder	SCID DSM-IV Diagnoses	55.20	82	39 (47.9%)	8
			Post-Vietnam veterans with Substance Use Disorder		46.76	236	131 (55.4%)	
			Persian Gulf veterans with Substance Use Disorder		34	55	33 (59.5%)	
Rehn, L. M. et al. (2009)(110)	Finland	Convenience Sampling (CS)	Male Finnish military conscripts	The Beck Depression Inventory (BDI)	20	126	4 (3.2%)	6
Rukskul, I. et al. (2009) (111)	Thailand	Convenience Sampling (CS)	Thai army personnel	Clinical Symptoms (Interviews)	45	1729	186 (10.75%)	5
Rukskul, I. (2010)(112)	Thailand	Convenience Sampling (CS)	Thai army personnel	Clinical Symptoms (Interviews)	45	213	7 (3.3%)	5
Fikretoglu, D. et al. (2010)(113)	Canada	Convenience Sampling (CS)	Canadian Community Health Survey-Canadian Forces KlineKline(CCHS-CF)	Diagnostic and Statistical Manual of Mental Disorders-IV (DSM-IV)	–	8441	1257 (14.9%)	8
Haskell, S. G. et al. (2010)(114)	USA	Convenience Sampling (CS)	War Veterans of Iraq and Afghanistan	Clinical Symptoms (Interviews)	32	Total (1229)	472 (38.4%)	7
					32	Male (1032)	380 (36.8%)	
					30	Female (197)	92 (46.7%)	
Luxton, D. D. et al. (2010)(115)	USA	Convenience Sampling (CS)	Active duty Soldiers between March 2006 and July 2009.	The PRIME-MD Patient Health Questionnaire (PHQ)	27.37	Total (6943)	704 (10.1%)	7
						Male (6427)	646 (10.0%)	
						Female (516)	58 (46.7%)	
Maguen, S. et al. (2010)(116)	USA	Convenience Sampling (CS)	Iraq and Afghanistan Veterans Enrolled in Veterans Affairs Health Care	Diagnostic and Statistical Manual of Mental Disorders-IV (DSM-IV)	31.21	Total (329049)	57,051 (17.33%)	8
					31.47	Male (288348)	47,876 (17%)	
					29.41	Female (40701)	9175 (23%)	
Stecker, T. et al. (2010)(117)	Lebanon	Convenience Sampling (CS)	Iraq/Afghanistan veterans	Diagnostic and Statistical Manual of Mental Disorders-IV (DSM-IV)	34.4	293,861	36,900 (12.5%)	6
			Iraq/Afghanistan Veterans with Alcohol Use Disorder			118,332	4568 (3.8%)	
Burnett-Zeigler, I. et al. (2011)(118)	USA	Random Sampling (CS)	Afghanistan and Iraq Veterans	The PRIME-MD Patient Health Questionnaire (PHQ)	–	362	64 (17.6%)	7
			Iraq/Afghanistan Veterans with Alcohol Use Disorder			200	72 (36%)	
Erbes, C. R. et al. (2011)(119)	USA	Convenience Sampling (CS)	National Guard/Reserve veterans returning from Iraq	The Beck Depression Inventory (BDI)	31.60	617	83 (13.5%)	7
Guerra, V. S. et al. (2011)(120)	USA	Convenience Sampling (CS)	Veterans in Operations Enduring Freedom and Iraqi Freedom (OEF/OIF)	The Beck Depression Inventory (BDI) Beck Scale for Suicide Ideation Scale for Suicide Ideation-Adapted	38.3	393	88 (22.4%)	8
Jakupcak, M. et al. (2011)(121)	USA	Convenience Sampling (CS)	Iraq and Afghanistan War Veterans in the U.S	The PRIME-MD Patient Health Questionnaire (PHQ)	31	336	126 (37.5%)	7
Kehle, S. M. et al. (2011)(122)	USA	Convenience Sampling (CS)	Soldiers from a National Guard Brigade Combat Team (BCT)	Diagnostic and Statistical Manual of Mental Disorders-IV (DSM-IV)	31.30	Total (348)	51 (15%)	7
						Male (304)	39 (13%)	
						Female (44)	12 (27%)	
			Alcohol use disorders			348	45 (13%)	
			Substance use disorders			348	4 (1%)	
Garber, B. G. et al. (2012)(123)	Canada	Convenience Sampling (CS)	Canadian Forces Members While on Deployment to Afghanistan	The PRIME-MD Patient Health Questionnaire (PHQ)	–	1572	73 (4.7%)	5
Maguen, S. et al. (2012)(124)	USA	Convenience Sampling (Re)	Iraq and Afghanistan Veterans	The Diagnostic and Statistical Manual-Fourth Edition (DSM-IV)	45	Total (74493)	41,424 (56%)	7
						Male (67238)	36,359 (54%)	
						Female (7255)	5065 (70%)	
Vasterling, J. J. et al. (2012)(125)	USA	Convenience Sampling (CS)	Iraq-deployed US Army soldiers	The Center for Epidemiological Studies Depression Scale (CES-D)	25.1	760	238 (31.3%)	7
Cohen, S. I. et al. (2013)(126)	USA	Convenience Sampling (CS)	US military veterans returning from Iraq and Afghanistan	The Diagnostic and Statistical Manual-Fourth Edition (DSM-IV)	–	93	44 (47.3%)	6
Harbertson, J. et al. (2013)(127)	USA	Convenience Sampling (CS)	Male Rwanda Defense Forces military personnel	The Center for Epidemiological Studies Depression Scale (CES-D)	30.9	1238	232 (22.1%)	7
						546	129 (23.7%)	
			Alcohol Use Disorder					
Marshall, B. D. et al. (2013)(128)	USA	Convenience Sampling (CS)	Ohio Army National Guard Soldiers	The PRIME-MD Patient Health Questionnaire (PHQ)	–	2117	128 (6%)	7
						142	17 (12%)	
			Soldiers with HIV					
Morrow, C. E. et al. (2013)(129)	USA	Convenience Sampling (CS)	U.S. Air Force	The PRIME-MD Patient Health Questionnaire (PHQ)	30.35	194	3 (1.6%)	5
Swinkels, C. M. et al. (2013)(130)	UK	Convenience Sampling (CS)	U.S. Afghanistan/Iraq Veterans	Diagnostic and Statistical Manual of Mental Disorders-IV (DSM-IV)	37.40	1640	308 (18.8%)	6
Chapman, P. L. et al. (2014)(131)	USA	Convenience Sampling (CS)	U.S. Army Combat Medics	The PRIME-MD Patient HealthQuestionnaire (PHQ)	43.54	543	73 (13.4%)	6
Clarke-Walper, K. et al. (2014)(132)	USA	Convenience Sampling (CS)	Soldiers who returned from Iraq or Afghanistan	The PRIME-MD Patient Health Questionnaire (PHQ)	–	7849	611 (8.1%)	7
						2328	304 (13.1%)	
			Alcohol use					
Curry, J. F. et al. (2014)(133)	USA	Convenience Sampling (CS)	Veterans	Diagnostic and Statistical Manual of Mental Disorders-IV (DSM-IV)	37.48	Total (1700)	652 (38.4%)	7
						Male (1354)	491 (36.3%)	
						Female (346)	161 (46.5%)	
			Veterans with alcohol use			623	72 (11.6%)	
			Veterans with substance use			154	7 (4.5%)	
Denneson, L. M. et al. (2014)(134)	USA	Convenience Sampling (CS)	Iraq and Afghanistan Veterans	The PRIME-MD Patient Health Questionnaire (PHQ)	–	465	237 (51%)	7
Don Richardson, J. et al. (2014)(135)	Canada	Convenience Sampling (CS)	Canadian Forces members and veterans	The PRIME-MD Patient Health Questionnaire (PHQ)	–	404	316 (78.2%)	7
Garber, B. G. et al. (2014)(136)	Canada	Random Sampling (CS)	Canadian armed forces personnel	The PRIME-MD Patient Health Questionnaire (PHQ)	–	16,153	593 (3.67%)	8
Heltemes, K. J. et al. (2014)(137)	USA	Random Sampling (CS)	Injured veterans	Diagnostic and Statistical Manual of Mental Disorders-IV (DSM-IV)	22.5	812	146 (18%)	6
Lehavot, K. et al. (2014)(138)	USA	Random Sampling (CS)	Sexual Minority and Heterosexual Women Veterans	The PRIME-MD Patient Health Questionnaire (PHQ)	48	697	260 (37.3%)	7
Ramsawh, H. J. et al. (2014)(139)	USA	Convenience Sampling (CS)	Active Duty Military Personnel	10-item Center for Epidemiologic Studies Depression Scale	35	5461	1914 (35%)	8
Bin Zubair, U. et al. (2015)(140)	Pakistan	Random Sampling (CS)	All military recruits were men and above the age of 17 years.	The Beck Depression Inventory (BDI)	20	313	159 (50.7%)	7
Cleveland, S. D. et al. (2015)(141)	USA	Convenience Sampling (CS)	Veterans and Civilian College Students	Diagnostic and Statistical Manual of Mental Disorders-IV (DSM-IV)	–	26,969	7982 (30.17%)	8
Foote, C. E. et al. (2015)(142)	USA	Random Sampling (CS)	Vietnam veterans	The PRIME-MD Patient Health Questionnaire (PHQ)	–	247	44 (17.8%)	7
Hamilton, A. B. et al. (2015) (143)	USA	Random Sampling (CS)	Employed Women Veterans	The five-question Mental Health Inventory (MHI-5)	–	1410	120 (4.1%)	7
						195	42 (27.3%)	
			Unemployed Women Veterans					
Hoerster, K. D. et al. (2015)(144)	USA	Random Sampling (CS)	Iraq and Afghanistan veterans	The PRIME-MD Patient Health Questionnaire (PHQ)	31.3	332	53 (16.3%)	7
Kim, N. Y. et al. (2015) (145)	USA	Convenience Sampling (CS)	Korean Soldiers	Scale for suicide ideation (SSI), The Beck Depression Inventory (BDI)	21.3	414	21 (5%)	6
Lundin, A. et al. (2015)(146)	Sweden	Random Sampling (CS)	Vietnam veterans	Diagnostic and Statistical Manual of Mental Disorders-IV (DSM-IV)	–	4251	1263 (29.7%)	7
McGuire, A. et al. (2015)(147)	UK	Random Sampling (CS)	Australian Defense Force (ADF men)	Diagnostic and Statistical Manual of Mental Disorders-IV (DSM-IV)	50	4091	454(11%)	7
			Department of Veterans’ Affairs (DVA women)			4761	869 (18.3%)	
Mysliwiec, V. et al. (2015)(148)	USA	Convenience Sampling (CS)	U.S. Military Personnel	Quick Inventory of Depressive Symptomatology (QIDS)	36.2	58	30 (51.7%)	7
Nasioudis, D. et al. (2015)(149)	Greece	Random Sampling (CS)	Greek military medicine cadets	The Zung Self-report Depression Scale (SDS)	19.84	Total (146)	57 (39%)	7
						Male (91)	36 (39.5%)	
						Female (55)	21 (38.2%)	
Vanderploeg, R. D. et al. (2015) (150)	USA	Convenience Sampling (CS)	Florida National Guard Members	The PRIME-MD Patient Health Questionnaire (PHQ)	–	3098	63 (2%)	7
Fink, D. S. et al. (2016) (151)	USA	Convenience Sampling (CS)	U.S. Army National Guard soldiers	Diagnostic and Statistical Manual of Mental Disorders-IV (DSM-IV)	44	671	154 (23%)	8
Forbes, D. et al. (2016)(152)	Australia	Convenience Sampling (CS)	Australian peacekeepers	Diagnostic and Statistical Manual of Mental Disorders-IV (DSM-IV)	46.5	2050	201 (9.8%)	8
Guloglu, B. et al. (2016)(153)	Turkey	Convenience Sampling (CS)	Turkish combat-injurednon-professional veterans	The Brief Symptom Inventory (BSI)	40	336	55 (16.4%)	7
Hardos, J. E. et al. (2016) (154)	USA	Convenience Sampling (CS)	Aircraft Maintenance Workers	The PRIME-MD Patient Health Questionnaire (PHQ)	29	4801	1042 (21.7%)	7
Herberman Mash, H. B. et al. (2016)(155)	USA	Convenience Sampling (CS)	U.S. Army soldiers	The 10-item Center for Epidemiologic Studies Depression Scale	–	3813	1368 (35.8%)	8
			U.S. Army soldiers with alcohol use			1210	583 (48.18%)	
Monteith, L. L. et al. (2016)(156)	USA	Convenience Sampling (CS)	Veterans	Beck Scale for Suicide Ideation (BSS), Multidimensional Suicide Inventory-28 (MSI) Negative Affect scale	49.6	Total (354)	169 (47.7%)	8
						Male (310)	146 (47.1%)	
						Female (44)	32 (52.3%)	
Phillips, K. M. et al. (2016)(157)	USA	Convenience Sampling (CS)	Iraq- and Afghanistan-era Veterans	20-item, self-report Center for Epidemiological Studies Depression Scale (CES-D)	35.1	359	108 (30%)	6
Zamorski, M. A. et al. (2016)(158)	Canada	Convenience Sampling (CS)	Canadian Armed Forces	Diagnostic and Statistical Manual of Mental Disorders-IV (DSM-IV)	–	5120	410 (8%)	7
Boakye, E. A. et al. (2017)(159)	USA	Random Sampling (CS)	Veterans	Self-Report	40	144	48 (33.3%)	7
			Veterans with alcohol use			75	24 (32%)	
Cohen, G. H. et al. (2017)(160)	USA	Convenience Sampling (CS)	Army National Guard Soldiers	The PRIME-MD Patient Health Questionnaire (PHQ), The PHQ-9 Item	–	1582	164 (10.3%)	8
			Army National with Alcohol Use			93	27 (29%)	
Gradus, J. L. et al. (2017)(161)	USA	Random Sampling (CS)	Veterans of the Iraq and Afghanistan Wars	20-item, self-report Center for Epidemiological Studies Depression Scale (CES-D), The 4-item Suicidal Behaviors Questionnaire-Short Form (SBQ-SF)	34	Total (2244)	712 (31.7%)	7
						Male (1062)	314 (29.5%)	
						Female (1099)	398 (36.3%)	
Packnett, E. R. et al. (2017)(162)	USA	Convenience Sampling (CS)	Army	Diagnostic and Statistical Manual of Mental Disorders-IV (DSM-IV)	–	34,487	1777 (5.1%)	8
			Navy			6602	263 (4%)	
			Marine Corps			8428	113 (1.3%)	
			Air Force			9510	729 (7.6%)	
Weeks, M. et al. (2017)(163)	Canada	Convenience Sampling (CS)	Canadian Military and Civilian Populations	Diagnostic and Statistical Manual of Mental Disorders-IV (DSM-IV)	35	6696	536 (8%)	8
Bartlett, B. A. et al. (2018)(164)	USA	Convenience Sampling (CS)	Military veterans	20-item, self-report Center for Epidemiological Studies Depression Scale (CES-D)	38.40	910	75 (9.8%)	6
Blakey, S. M. et al. (2018)(165)	USA	Convenience Sampling (CS)	U.S. veterans, active duty personnel, and National Guard and Reserve members	Diagnostic and Statistical Manual of Mental Disorders-IV (DSM-IV)	37.8	667	169 (25.3%)	7
Boulos, D. et al. (2018)(166)	Canada	Random Sampling (CS)	Regular Force personnel	Diagnostic and Statistical Manual of Mental Disorders-IV (DSM-IV)	–	3385	129 (3.8%)	7
			Reserve Force personnel			1469	55 (3.7%)	
Dillon, K. H. et al. (2018)(167)	USA	Convenience Sampling (CS)	Iraq/Afghanistan-era veterans	The Beck Scale for Suicide Ideation (BSS), The Structured Clinical Interview for DSM-IV-TR (SCID)	–	3238	1315 (40.6%)	7
Don Richardson, J. et al. (2018)(168)	Canada	Convenience Sampling (CS)	Canadian Armed Forces members and veterans	The PRIME-MD Patient Health Questionnaire (PHQ)	44.6	522	413 (79.1%)	7
Elbogen, E. B. et al. (2018)(169)	USA	Convenience Sampling (CS)	Iraq/Afghanistan-era veterans	Diagnostic and Statistical Manual of Mental Disorders-IV (DSM-IV)	34.9	1172	375 (32%)	6
Hourani, L. L. et al. (2018)(170)	USA	Convenience Sampling (CS)	Active duty military personnel	The PRIME-MD Patient Health Questionnaire (PHQ), Checklist	–	947	115 (15.4%)	7
Kizilhan, J. I. et al. (2018)(171)	Iraq	Convenience Sampling (CS)	Child soldiers	Diagnostic and Statistical Manual of Mental Disorders-IV (DSM-IV)	12.6	81	37 (45.6%)	6
McDonald, S. D. et al. (2018)(172)	USA	Convenience Sampling (CS)	U.S. Department of Veterans Affairs outpatients	Diagnostic and Statistical Manual of Mental Disorders-IV (DSM-IV)	58.1	280	53 (19%)	7
Stefanovics, E. A. et al. (2018)(173)	USA	Convenience Sampling (CS)	US Veterans	The Patient Health Questionnaire-4	59	3122	209 (6.7%)	7
Vun, E. et al. (2018)(174)	Canada	Convenience Sampling (CS)	Canadian Armed Forces active personnel	Diagnostic and Statistical Manual of Mental Disorders-IV (DSM-IV)	35.4	6696	517 (8%)	8
Waitzkin, H. et al. (2018)(175)	USA	Convenience Sampling (CS)	Military Personnel	The PRIME-MD Patient Health Questionnaire (PHQ)	–	198	143 (72%)	7
Byrne, S. P. et al. (2019)(176)	USA	Convenience Sampling (CS)	U.S. military veterans	The PRIME-MD Patient Health Questionnaire (PHQ)	53.4	158	62 (34.7%)	7
Carney, B. et al. (2019)(177)	USA	Random Sampling (CS)	US Military population with HIV infection	20- item, self-report Center for Epidemiological Studies Depression Scale (CES-D)	32	662	114 (17.2%)	8
Jones, N. et al. (2019)(178)	UK	Random Sampling (CS)	UK Armed Forces	The PRIME-MD Patient Health Questionnaire (PHQ)	–	Total (1448)	110 (7.6%)	6
						Male (1229)	93 (7.7%)	
						Female (219)	17 (7.9%)	
Lucas, C. L. et al. (2019)(179)	USA	Convenience Sampling (CS)	Military Personnel	The PRIME-MD Patient Health Questionnaire (PHQ)	–	Total (1980)	660 (37.9%)	7
						Male (1665)	530 (36.2%)	
						Female (315)	130 (46.8%)	
Nichter, B. et al. (2019)(180)	USA	Random Sampling (CS)	U.S. veteran population	The Patient Health Questionnaire-4 (PHQ-4), The Patient Health Questionnaire-9 (PHQ-9)	60.3	2732	201 (7.3%)	9
Start, A. R. et al. (2019)(181)	USA	Convenience Sampling (CS)	Military Personnel	The Patient Health Questionnaire-9 (PHQ-9)	–	944	72 (7.6%)	7
Blosnich, J. R. et al. (2020)(182)	USA	Random Sampling (CS)	Military Veterans	Diagnostic and Statistical Manual of Mental Disorders-IV (DSM-IV)	–	293,872	45,391 (15.4%)	9
Forys-Donahue, K. L. et al. (2020)(183)	USA	Random Sampling (CS)	US Army population	The Patient Health Questionnaire-9 (PHQ-9)	–	7043	774 (11%)	6
Gjerstad, C. L. et al. (2020)(184)	Norway	Convenience Sampling (CS)	Norwegian Peacekeepers	The Hospital Anxiety and Depression Scale (HADS)	30	10,450	417 (4%)	8
Groll, D. L. et al. (2020)(185)	Canada	Convenience Sampling (CS)	Canadian military persons	The Patient Health Questionnaire-9 (PHQ-9)	–	477	61 (12.8%)	8
Gross, G. M. et al. (2020)(186)	USA	Random Sampling (CS)	U.S. veteran population	The Patient Health Questionnaire-9 (PHQ-9)	35	Total (650)	306 (47%)	7
						Male (498)	192 (38.6%)	
						Female (352)	114 (32.4%)	
Shim, E. J. et al. (2020)(187)	Korea	Random Sampling (CS)	Korean military population	The Mini International Neuropsychiatric Interview Plus (MINI-Plus), The Patient Health Questionnaire-9 (PHQ-9)	50.6	1937	162 (8.4%)	8
Smigelsky, M. A. et al. (2020)(188)	USA	Convenience Sampling (CS)	U.S. military population	Diagnostic and Statistical Manual of Mental Disorders-IV (DSM-IV)	37.6	1002	210 (21%)	6
Smith, L. M. et al. (2020)(189)	USA	Convenience Sampling (CS)	U.S. Air Force Basic Military Training	The Patient Health Questionnaire-9 (PHQ-9)	–	85	20 (23.5%)	5
Stefanovics, E. A. et al. (2020)(190)	USA	Convenience Sampling (CS)	U.S. Military Veterans	The Mini International Neuropsychiatric Interview (MINI), The Patient Health Questionnaire-9 (PHQ-9)	55	1308	340 (30%)	5
Taillieu, T. L. et al. (2020)(191)	Canada	Convenience Sampling (CS)	Canadian Armed Forces	Diagnostic and Statistical Manual of Mental Disorders-IV (DSM-IV)	–	6447	1006(15.6%)	5
Wang, J. et al. (2020)(192)	USA	Convenience Sampling (CS)	U.S. Reserve and National Guard Personnel	The Patient Health Questionnaire-9 (PHQ-9)	34.4	3503	86 (2.5%)	6
Ursano, R. J. et al. (2020)(193)	USA	Convenience Sampling (CS)	US Army Soldiers During Deployment in Afghanistan	Diagnostic and Statistical Manual of Mental Disorders-IV (DSM-IV)	–	3957	173 (4.1%)	7
Yeom, C. W. et al. (2020)(194)	Korea	Convenience Sampling (CS)	Korean military personal	The Mini International Neuropsychiatric Interview Plus (MINI-Plus Suicidality module), The Patient Health Questionnaire-9 (PHQ-9)	21.4	480	27(5.6%)	6

**Table 2:** The study characteristics of included studies about suicide attempted and thought

Authors (Years)	Country	Type of Sampling (Type of Study)	Study Population	Depression Assessment Method	Age (Mean)	Sample size	Prevalence of Suicide (%)		NOS Score
							Attempts	Thoughts	
Helzer, J. E. et al. (1976) (66)	USA	Random Sampling (CS)	Army men	Clinical Symptoms (Interviews)	–	470	NR	42 (9%)	7
Bohnker, B. et al. (1992) (195)	USA	Random Sampling (CS)	Aircraft Carrier (men)	NR	–	150	102 (68%)	NR	6
Brown, G. R. et al. (1993) (70)	USA	Random Sampling (CS)	Air Forces men with HIV	Structured Interview Guide for the Hamilton Anxiety and Depression Scales (SIGH-AD)	35	442	24 (5.4%)	NR	8
Lish, J. D. et al. (1996) (74)	USA	Random Sampling (CS)	Army men and women	Brief self-report questionnaire (SCRENNER)	21.2	669	NR	51 (7.62%)	7
Benda, B. B. (2003) (196)	USA	Convenience Sampling (CS)	Veterans Who Abuse Substances	Multi-Problem Screening Inventory (MPSI)	50.3	600	240 (40%)	184 (30.7%)	7
Ritchie, E. C. et al. (2003)(197)	USA	Convenience Sampling (CS)	Men and Women in the Navy and Marine Corps	–	43	100	54 (54%)	NR	5
Benda, B. B. et al. (2005)(198)	USA	Convenience Sampling (CS)	Veterans Who Abuse Substances	The Multi-Problem Screening Inventory (MPSI)	40.3	625	197 (31.5%)	291 (46.5%)	6
Hoge, C. W. et al. (2006)(101)	USA	Random Sampling (CS)	Army soldiers and Marines	The PRIME-MD Patient Health Questionnaire (PHQ)	31.2	303,905	NR	3501 (1.15%)	8
Dove, M. B. et al. (2007)(104)	USA	Convenience Sampling (CS)	Women Entering a Military Substance Use Disorder	Depression Checklist	–	86	NR	15 (17.4%)	5
Kline, A. et al. (2009)(109)	USA	Convenience Sampling (CS)	Vietnam veterans with Substance Use Disorder	SCID DSM-IV Diagnoses	55.20	82	23 (27.8%)	5 (6.1%)	8
			Post-Vietnam veterans with Substance Use Disorder		46.76	236	63 (26.8%)	16 (6.8%)	
			Persian Gulf veterans with Substance Use Disorder		34	55	9 (15.4%)	5 (9.1%)	
Rehn, L. M. et al. (2009)(110)	Finland	Convenience Sampling (CS)	Male Finnish military conscripts	The Beck Depression Inventory (BDI)	20	126	NR	9 (7.1%)	6
Belik, S. L. et al. (2010)(199)	Canada	Convenience Sampling (CS)	The Canadian Forces	Diagnostic and Statistical Manual of Mental Disorders-IV (DSM-IV)	–	37,129	236 (0.8%)	1613 (4.34%)	8
Guerra, V. S. et al. (2011)(120)	USA	Convenience Sampling (CS)	Veterans in Operations Enduring Freedom and Iraqi Freedom (OEF/OIF)	The Beck Depression Inventory (BDI) Beck Scale for Suicide Ideation Scale for Suicide Ideation-Adapted	38.3	393	34 (8.7%)	45 (11.5%)	8
Mansfield, A. J. et al. (2011)(200)	USA	Convenience Sampling (CS)	Military Personnel	The Center for Epidemiological Studies Depression (CES-D) scale, The PRIME-MD Patient Health Questionnaire (PHQ)	28.1	3069	NR	215 (7%)	6
			Military Personnel (Navy)		31.8	1843		98 (5.3%)	
					25.8	1226		110 (9%)	
			Military Personnel (Marine)						
						Female (7255)			
Maguen, S. et al. (2012)(201)	USA	Convenience Sampling (CS)	Vietnam veterans	Checklist	40	244	12 (4.9%)	40 (16.4%)	6
Swinkels, C. M. et al. (2013)(130)	UK	Convenience Sampling (CS)	U.S. Afghanistan/Iraq Veterans	Diagnostic and Statistical Manual of Mental Disorders-IV (DSM-IV)	37.40	1640	132 (8%)	NR	6
Bryan, C. J. et al. (2013)(202)	USA	Convenience Sampling (CS)	Deployed Military Personnel	The 4-item Suicidal Behaviors Questionnaire– Revised (SBQ-R)	–	161	NR	35 (21.7%)	5
Bryan, C. J. et al. (2013)(203)	USA	Convenience Sampling (CS)	Deployed Military Personnel	The 4-item Suicidal Behaviors Questionnaire– Revised (SBQ-R)	–	158	3 (1.5%)	21 (13.1%)	5
Bryan, C. J. et al. (2013)(204)	USA	Convenience Sampling (CS)	Air Force Personnel	Beck Scale for Suicidal Ideation-Current (BSSI-C)	25.9	273	NR	53 (19.4%)	5
Bryan, C. J. et al. (2013)(205)	USA	Convenience Sampling (CS)	Deployed Military Personnel	The Self-Injurious Thoughts and Behaviors Interview (SITBI)	34.2	69	NR	25 (36.2%)	5
Blosnich, J. R. et al. (2014)(206)	USA	Convenience Sampling (CS)	Deployed Military Personnel	Checklist	–	4250	NR	154 (3.3%)	5
Bryan, C. J. et al. (2014)(207)	USA	Convenience Sampling (CS)	Deployed Military Personnel	The Self-Injurious Thoughts and Behaviors Interview (SITBI)	36.7	374	29 (7.8%)	136 (36.4%)	6
Mash, H. B. et al. (2014)(208)	USA	Convenience Sampling (CS)	US Army	Checklist	–	4999	NR	303(6%)	6
Don Richardson, J. et al. (2014)(135)	Canada	Convenience Sampling (CS)	Canadian Forces members and veterans	The PRIME-MD Patient Health Questionnaire (PHQ)	–	404	NR	68 (16.8%)	7
Ramsawh, H. J. et al. (2014)(139)	USA	Convenience Sampling (CS)	Active Duty Military Personnel	10-item Center for Epidemiologic Studies Depression Scale	35	5461	346 (6.33%)	NR	8
Bryan, C. J. et al. (2015)(209)	USA	Convenience Sampling (CS)	Air Force personnel	The Suicidal Behaviors Questionnaire Revised (SBQ-R)	–	168	2 (1.2%)	29 (17.3%)	7
Cleveland, S. D. et al. (2015)(141)	USA	Convenience Sampling (CS)	Veterans and Civilian College Students	Diagnostic and Statistical Manual of Mental Disorders-IV (DSM-IV)	–	26,969	282 (1.07%)	1730 (6.54%)	8
Kim, N. Y. et al. (2015) (145)	USA	Convenience Sampling (CS)	Korean Soldiers	Scale for suicide ideation (SSI), The Beck Depression Inventory (BDI)	21.3	414	NR	80 (19.3%)	6
Ursano, R. J. et al. (2015)(210)	USA	Convenience Sampling (CS)	Soldiers	The Columbia Suicidal Severity Rating Scale (C-SSRS)	20	38,237	536 (1.9%)	5353 (14%)	8
Vanderploeg, R. D. et al. (2015) (150)	USA	Convenience Sampling (CS)	Florida National Guard Members	The PRIME-MD Patient Health Questionnaire (PHQ)	–	3098	NR	130 (4.2%)	7
Forbes, D. et al. (2016)(152)	Australia	Convenience Sampling (CS)	Australian peacekeepers	Diagnostic and Statistical Manual of Mental Disorders-IV (DSM-IV)	46.5	2050	25 (1.2%)	275 (13.4%)	8
Herberman Mash, H. B. et al. (2016)(155)	USA	Convenience Sampling (CS)	U.S. Army soldiers	The 10-item Center for Epidemiologic Studies Depression Scale	–	3813	230 (6%)	NR	8
			U.S. Army soldiers with alcohol use			1210	100 (8.3%)		
Monteith, L. L. et al. (2016)(156)	USA	Convenience Sampling (CS)	Veterans	Beck Scale for Suicide Ideation (BSS), Multidimensional Suicide Inventory-28 (MSI) Negative Affect scale	49.6	Total (354)	92 (26.8%)	NR	8
						Male (310)	82 (26.5%)		
						Female (44)	13 (29.5%)		
Cohen, G. H. et al. (2017)(160)	USA	Convenience Sampling (CS)	Army National Guard Soldiers	The PRIME-MD Patient Health Questionnaire (PHQ), The PHQ-9 Item	–	1582	NR	42 (2.6%)	8
			Army National with Alcohol Use			93		8 (8.6%)	
Gradus, J. L. et al. (2017)(161)	USA	Random Sampling (CS)	Veterans of the Iraq and Afghanistan Wars	20- item, self-report Center for Epidemiological Studies Depression Scale (CES-D), The 4-item Suicidal Behaviors Questionnaire-Short Form (SBQ-SF)	34	Total (2244)	NR	370 (16.5%)	7
						Male (1062)		179 (16.9%)	
						Female (1099)		191 (17.4%)	
Weeks, M. et al. (2017)(163)	Canada	Convenience Sampling (CS)	Canadian Military and Civilian Populations	Diagnostic and Statistical Manual of Mental Disorders-IV (DSM-IV)	35	6696	NR	289 (4. %)	8
Bartlett, B. A. et al. (2018)(164)	USA	Convenience Sampling (CS)	Military veterans	20- item, self-report Center for Epidemiological Studies Depression Scale (CES-D)	38.40	910	62 (7.5%)	NR	6
Boulos, D. et al. (2018)(166)	Canada	Random Sampling (CS)	Regular Force personnel	Diagnostic and Statistical Manual of Mental Disorders-IV (DSM-IV)	–	3385	NR	156 (4.6%)	7
			Reserve Force personnel			1469		82 (5.6%)	
Dillon, K. H. et al. (2018)(167)	USA	Convenience Sampling (CS)	Iraq/Afghanistan-era veterans	The Beck Scale for Suicide Ideation (BSS), The Structured Clinical Interview for DSM-IV-TR (SCID)	–	3238	291 (9%)	NR	7
Elbogen, E. B. et al. (2018)(169)	USA	Convenience Sampling (CS)	Iraq/Afghanistan-era veterans	Diagnostic and Statistical Manual of Mental Disorders-IV (DSM-IV)	34.9	1172	87 (7.5%)	NR	6
Hourani, L. L. et al. (2018)(170)	USA	Convenience Sampling (CS)	Active duty military personnel	The PRIME-MD Patient Health Questionnaire (PHQ), Checklist	–	947	16 (2.1%)	71 (9.2%)	7
Kachadourian, L. K. et al. (2018)(211)	USA	Convenience Sampling (CS)	Veterans	The Columbia-Suicide Severity Rating Scale (C-SSRS)	43.9	93	19 (21.6%)	NR	6
Kerr, K. et al. (2018)(212)	Australia	Convenience Sampling (CS)	Australian veterans	Checklist	54.6	229	54 (23.6%)	NR	6
Waitzkin, H. et al. (2018)(175)	USA	Convenience Sampling (CS)	Military Personnel	The PRIME-MD Patient Health Questionnaire (PHQ)	–	198	NR	92 (48%)	7
Byrne, S. P. et al. (2019)(176)	USA	Convenience Sampling (CS)	U.S. military veterans	The PRIME-MD Patient Health Questionnaire (PHQ)	53.4	158	40 (24.4%)	39 (30.2%)	7
Nichter, B. et al. (2019)(180)	USA	Random Sampling (CS)	U.S. veteran population	The Patient Health Questionnaire-4 (PHQ-4), The Patient Health Questionnaire-9 (PHQ-9)	60.3	2732	134 (4.9%)	248 (9%)	9
Start, A. R. et al. (2019)(181)	USA	Convenience Sampling (CS)	Military Personnel	The Patient Health Questionnaire-9 (PHQ-9)	–	944	NR	31 (3.3%)	7
Blosnich, J. R. et al. (2020)(182)	USA	Random Sampling (CS)	Military Veterans	Diagnostic and Statistical Manual of Mental Disorders-IV (DSM-IV)	–	293,872	1035 (0.3%)	2999 (1%)	9
Cramer, R. J. et al. (2020)(213)	USA	Random Sampling (CS)	Military Personnel	The Suicide Behaviors Questionnaire-Revised (SBQ-R)	–	200	96 (48%)	NR	6
Groll, D. L. et al. (2020)(185)	Canada	Convenience Sampling (CS)	Canadian military persons	The Patient Health Questionnaire-9 (PHQ-9)	–	477	19 (4%)	76 (16%)	8
Shim, E. J. et al. (2020)(187)	Korea	Random Sampling (CS)	Korean military population	The Mini International Neuropsychiatric Interview Plus (MINI-Plus), The Patient Health Questionnaire-9 (PHQ-9)	50.6	1937	87 (4.5%)	NR	8
Smigelsky, M. A. et al. (2020)(188)	USA	Convenience Sampling (CS)	U.S. military population	Diagnostic and Statistical Manual of Mental Disorders-IV (DSM-IV)	37.6	1002	41 (4%)	NR	6
Stefanovics, E. A. et al. (2020)(190)	USA	Convenience Sampling (CS)	U.S. Military Veterans	The Mini International Neuropsychiatric Interview (MINI), The Patient Health Questionnaire-9 (PHQ-9)	55	1308	118 (9%)	165(12.6%)	5
Wang, J. et al. (2020)(192)	USA	Convenience Sampling (CS)	U.S. Reserve and National Guard Personnel	The Patient Health Questionnaire-9 (PHQ-9)	34.4	3503	NR	101 (2.9%)	6
Anestis, M. D. et al. (2020)(214)	USA	Convenience Sampling (CS)	U.S. Military Veterans	The Suicide Behaviors Questionnaire-Revised (SBQ-R)	27.0	953	NR	105 (15.2%)	5
Monteith, L. L. et al. (2020)(215)	USA	Convenience Sampling (CS)	Female veterans	Checklist	55.6	439	158(36%)	113(25.7%)	5
Ursano, R. J. et al. (2020)(193)	USA	Convenience Sampling (CS)	US Army Soldiers During Deployment in Afghanistan	Diagnostic and Statistical Manual of Mental Disorders-IV (DSM-IV)	–	3957	NR	85 (2.1%)	7
Yeom, C. W. et al. (2020)(194)	Korea	Convenience Sampling (CS)	Korean military personal	The Mini International Neuropsychiatric Interview Plus (MINI-Plus Suicidality module), The Patient Health Questionnaire-9 (PHQ-9)	21.4	480	22(4.5%)	NR	6

### Quantitative analysis

#### Prevalence of depression in the all military

Initially, the studies were divided into two groups: the active duty military community and the veteran’s community in terms of the study population. Then, separate analyzes were performed for each of these communities and the prevalence of depression in each was meta-analyzed. Of the 133 final selected cross-sectional studies, 80 were in the veterans and 100 were in the active duty military personnel.

#### Prevalence of depression in the active duty military

In these studies, 1,278,837 employees of the active or serving military had been examined, of whom 273,173 had depression. After combining the results of these studies, the overall pooled prevalence of depression in the active or in-service military was 23% with a confidence interval of 20 to 26%. The percentage of heterogeneity was 99.91% which was statistically significant (Table 3).

**Table 3:** The pooled estimate of prevalence of depression in active duty and veteran military

Categories	No. of Studies (Sample Size)	Pooled Prevalence (% 95 CI)	Between studies heterogeneity assessment (%)			Between subgroups heterogeneity assessment (%)	
			*I*^*2*^	*P*_*Heterogenity*_	*Z*	Q	*P*_*Heterogenity*_
The prevalence of depression in active duty military							
Total	100 (1278837)	23% (20–26%)	87.91%	0.018	27.74	–	–
Sampling Method							
Convinces Sampling	67 (939796)	21% (18–25%)	66.90%	0.030	20.25	9.33	0.001
Random Sampling	33 (339041)	26% (19–32%)	54.80%	0.050	13.11		
Type of Forces							
Air Forces	5 (4562)	20% (9–33%)	83.93%	0.040	5.59	8.98	0.001
Armed Forces	36 (995073)	22% (20–23%)	89.45%	0.034	20.05		
Marine Forces	6 (775778)	31% (16–48%)	90.86%	0.0001	6.22		
Military Forces	53 (201624)	22% (16–28%)	79.89%	0.005	12.41		
Population Healthy Forces	90 (1152451)	22% (20–25%)	99.87%	0.0001	18.28	10.03	0.001
Forces with HIV/AIDS	3 (113620)	15% (3–36%)	–	–	3.16		
Forces with Alcohol Use	5 (8303)	29% (13–47%)	99.96%	0.0001	5.70		
Forces with Substance Use	2 (4463)	37%(36–39%)	–	–	18.04		
Gender							
Total	71 (1163273)	22% (20–25%)	90.88%	0.0001	20.19	10.01	0.001
Male	20 (110847)	23% (12–37%)	91.83%	0.0001	6.45		
Female	9 (4717)	25% (13–40%)	89.99%	0.012	6.15		
Tools							
BDI Scale	9 (38888)	25% (15–36%)	65.75%	0.054	5.88	5.09	0.001
CES-D Scale	7 (15365)	13% (8–19%)	50.20%	0.130	4.05		
Interviews	13 (16980)	25% (17–35%)	67.38%	0.060	10.22		
DSM-IV Scale	36 (202430)	15% (11–19%)	60.07%	0.078	12.41		
BSI Scale	1 (236)	56% (49–60%)	–	–	–		
HAMD Scale	1 (197)	47% (40–54%)	–	–	–		
HADS Scale	1 (6943)	10% (9–11%)	–	–	–		
PHQ Scale	24 (692087)	15% (13–17%)	78.62%	0.059	9.32		
SDS Scale	5 (304767)	20% (14–26%)	72.99%	0.059	9.03		
Country							
Canada	10 (318747)	21% (16–26%)	49.46%	0.760	4.99		
Korea	2 (430)	20% (16–24%)	0.0%	0.782	1.49		
Thailand	2 (2272)	39% (37–41%)	0.0%	0.800	0.98	17.74	0.001
United Kingdom	6 (2034)	32% (10–59%)	54.32%	0.763	4.05		
USA	66 (929016)	21% (17–25%)	78.96%	0.050	13.54		
Greece	3 (6845)	20% (1–52%)	0.0%	0.980	2.43		
The prevalence of depression in veteran military							
Total	80 (887982)	20% (18–22%)	79.80%	0.032	31.46	–	–
Sampling Method							
Convinces Sampling	55 (565979)	19% (16–21%)	69.78%	0.049	17.25	2.12	0.150
Random Sampling	25 (322003)	22% (18–27%)	58.26%	0.054	10.02		
Type of Forces							
Air Forces	NR	–	–	–	–		
Armed Forces	68 (583048)	19% (17–22%)	76.97%	0.054	16.88	1.27	0.260
Marine Forces	NR	–	–	–	–		
Military Forces	12 (304934)	24% (16–33%)	64.66%	0.034	9.83		
Population							
Healthy Forces	64 (856091)	19% (17–22%)	99.09%	0.0001	18.28	28.40	0.001
Forces with HIV/AIDS	2 (1257)	16% (14–18%)	91.33%	0.0001	22.32		
Forces with Alcohol Use	4 (1780)	29% (21–37%)	98.44%	0.0001	11.92		
Forces with Substance Use	4 (4397)	10%(6–14%)	74.50%	0.0001	8.68		
Forces with HCV	6 (24457)	29% (17–43%)	88.93	0.001	7.36		
Gender							
Total	55 (237654)	20% (17–23%)	90.88%	0.0001	22.36	0.12	0.873
Male	15 (343584)	21% (13–31%)	91.91%	0.0001	7.75		
Female	10 (306744)	20% (14–26%)	88.49%	0.0001	11.40		
Tools							
BDI Scale	7 (415692)	14% (9–21%)	55.15%	0.060	7.97		
CES-D Scale	11 (318802)	18% (13–25%)	40.45%	0.761	10.80		
Interviews	13 (50675)	20% (11–31%)	60.22%	0.181	6.74		
DSM-IV Scale	11 (64263)	15% (9–22%)	78.99%	0.028	7.54		
PHQ Scale	29 (28445)	21% (17–25%)	78.48%	0.049	17.52		
SDS Scale	2 (1300)	47% (44–50%)	52.04%	0.059	9.14		
GDS Scale	1 (1032)	37% (34–40%)		–	20.91		
MHI Scale	4 (3649)	35% (15–59%)	−50.74%	0.601	4.91		
QIDS Scale	1 (1002)	21% (18–24%)	–	–	19.13		
HDRS Scale	1 (3122)	7% (6–8%)	–	–	18.28	22.16	0.001
Country							
Canada	2 (2365)	13% (10–15%)	0.0%	0.880	3.85		
Croatia	2 (118669)	4% (6–8%)	0.0%	0.893	4.91	31.46	0.001
USA	70 (733009)	20% (18–22%)	67.84%	0.049	29.94		

Beck Depression Inventory (BDI), Center for Epidemiological Studies Depression (CES-D), Clinical Symptoms (Interviews), Diagnostic and Statistical Manual of Mental Disorders-IV (DSM-IV), The Brief Symptom Inventory (BSI), The Hamilton Depression Rating Scale (HAMD), The Hospital Anxiety and Depression Scale (HADS), The Patient Health Questionnaire (PHQ), The Zung Self-Report Depression Scale (SDS), Geriatric Depression Scale (GDS), Mental Health Inventory (MHI), Quick Inventory of Depressive Symptomatology (QIDS), Hamilton Depression Rating Scale (HDRS)-24 item

The pooled prevalence of depression was 21% (% 95 CI; 18–25%) in studies where the sampling method was the available one (convinces sampling). A total of 67 studies used this type of sampling method, which had examined a total of 939,796 active members, of whom 21,7487 had been considered depressed. In addition, 33 studies with a sample size of 339,041 people had used the random sampling method to collect their samples. After combining these studies, the pooled prevalence of depression was estimated to be 26% (% 95 CI; 19–32%) (Table 3).

In this meta-analysis, the pooled prevalence of depression in active duty military personnel was also calculated based on the location and the results were reported in Table 3. The results showed that the pooled prevalence of depression in active air, land, and naval forces was 20% (% 95 CI; 9–33%), 22% (% 95 CI; 20–23%), and 31% (% 95 CI; 16–48%), respectively. In 53 cross-sectional studies, it had not been specified that in which military unit, the study population was serving and it had been mentioned as military forces in that studies, so, a group called military forces was formed, the sample size of which was equal to 201,624 active military personnel of whom 65,158 people were depressed. The pooled prevalence of depression after a combination of these studies was 22% (% 95 CI; 16–28%) (Table 3).

The pooled prevalence of depression in active militaries with HIV was 15% (% 95 CI; 3–36%), in active militaries with substance use was 37% (% 95 CI; 36–39%), in militaries using alcohol was equal to 29% (% 95 CI; 13–47%) and finally in healthy and disease-free military members was equal to 22% (% 95 CI; 20–25%) (Table 3).

The pooled prevalence of depression in the active military varied by gender. A total of 71 cross-sectional studies had not identified the gender of the study population while 20 and 9 studies had been performed on military men and women, respectively. In studies that had not specified gender, the sample size was 1,163,273 people, of whom 221,910 individuals were depressed. The sample size in cross-sectional studies on military men and women was 110,847 and 4717 people, respectively, of whom 50,370 and 893 were depressed, respectively. The results of meta-analysis showed that the pooled prevalence of depression in male soldiers was equal to 23% (% 95 CI; 12–37%) while in military women, it was equal to 25% (% 95 CI; 31–40%) (Table 3).

Thirty-six cross-sectional studies had used the diagnostic and statistical manual of mental disorders-IV (DSM-IV), 24 studies had used the patient health questionnaire (PHQ), 13 studies had applied interviews using clinical criteria and symptoms, 5 studies had applied the Zung self-tool report depression scale (SDS), 9 studies had used the beck depression inventory (BDI) and 7 studies had used the center for epidemiological studies depression (CES-D) to diagnose depression in the active or in-service military. The overall prevalence of depression according to the diagnostic and statistical manual of mental disorders-IV (DSM-IV) was 15% (% 95 CI; 17–35%), according to the patient health questionnaire (PHQ), it was 15% (% 95 CI; 13–17%), And according to the Zung self-report depression scale (SDS), it was equal to 20% (% 95 CI; 14–26%). Also, the overall pooled prevalence based on beck depression inventory (BDI) and the center for epidemiological studies depression (CES-D) was 25% (% 95 CI; 15–36%) and 13% (% 95 CI; 8–19%), respectively (Table 3).

In the case of the active military, subgroup results by country showed most studies had been conducted in the United States that after combining 66 studies conducted in this country, the prevalence of depression was 21% (with a confidence interval of 17 to 25%). The prevalence of depression in Thailand and the UK, which was 39 and 30%, respectively, was higher than that in other countries. The rest of the studies had been individually conducted in only one country and because of their number of primary studies not be used for meta-analysis (Table 3).

#### Publication bias, and meta-regression in studies related to the active military

**Fig. 2:**
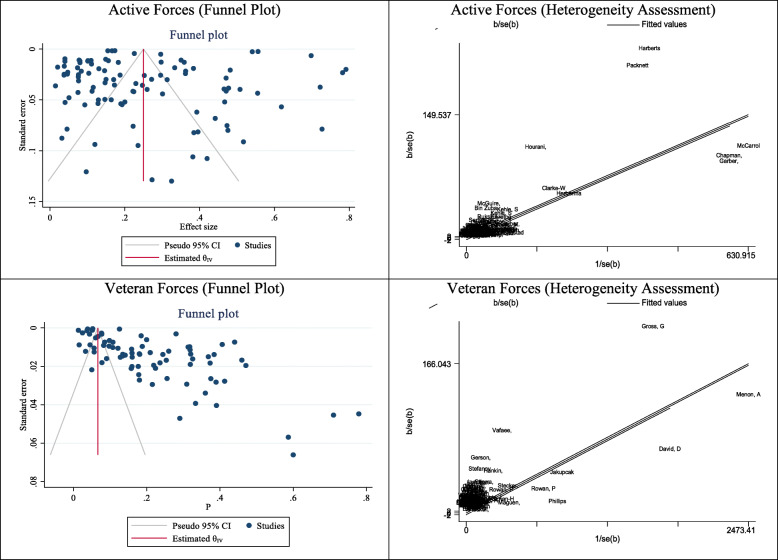
Results of Publication bias and Heterogeneity in pooled prevalence of depression in active duty and veteran military

The results of the publication bias were shown in Fig. 2 for studies related to the active military. The results of the Eggers test showed that diffusion bias did not occur in calculating the prevalence of depression in the active military (B: 0.96, SE: 0.69, P: 0.167) (Fig. 2). In meta-regression analysis, the effect of military personnel age on prevalence was studied and analyzed. The results presented that age had a significant effect on the prevalence of depression in the active military and for every 1 year of age, depression increased by 0.04%. The results of heterogeneity evaluation demonstrated that 5 studies were the cause of heterogeneity in the meta-analysis of the depression prevalence in active military (Fig. 2).

#### Prevalence of depression in veterans

Regarding the prevalence of depression in veterans, 80 cross-sectional articles with a sample size of 887,982 people were reviewed, of whom 822,967 people were depressed. After combining the results of these studies, the overall pooled prevalence of depression in veterans was 20% (% 95 CI; 18–22%). The percentage of heterogeneity was 99.80% which was statistically significant (Table 3).

The results of the subgroup analysis showed that 55 studies had used the convinces sampling method and 25 studies had used the random sampling method to determine the prevalence of depression in veterans. The sample size in the studies that had used the convinces sampling method was equal to 565,979 people. After combining their results, the pooled prevalence of depression was equal to 19% (% 95 CI; 16–21%). Also, the sample size in studies that had used the random sampling method was equal to 32,2003 people. After combining their results, the pooled prevalence of depression in veterans was equal to 22% (% 95 CI; 18–27%) (Table 3).

Regarding the military community of different divisions, the analysis showed that in the case of veterans, 68 studies had been conducted in the veterans’ community of the Army, and 12 studies had been conducted in the entire military (without separating the different divisions). There was no study in the Air Force or Navy. The sample size in military veterans was 583,048 people and after combining these results, the pooled prevalence of depression was 19% (% 95 CI; 17–22%) (Table 3).

The results of meta-analysis based on questionnaires and various measurement tools showed that heterogeneity of pooled prevalence was significantly reduced. In this section, 7 cross-sectional studies included in the meta-analysis using the beck depression inventory (BDI) questionnaire, 11 studies using the center for epidemiological studies depression (CES-D), 13 studies based on clinical criteria and interviews, 11 studies based on diagnostic and statistical manual of mental disorders-IV (DSM-IV), 29 studies based on the patient health questionnaire (PHQ), 2 studies based on the Zung self-report depression scale (SDS), 4 studies based on mental health inventory (MHI), 1 study based on Hamilton depression rating scale (HDRS), 1 study based on the quick inventory of depressive symptomatology (QIDS), and 1 study based on the geriatric depression scale (GDS) had examined depression in veterans. The results of the meta-analysis showed that the prevalence of depression according to the statistical manual of mental disorders-IV (DSM-IV), the patient health questionnaire (PHQ), and beck depression inventory (BDI) was 15% (% 95 CI; 9–22%), 21% (% 95 CI; 17–25%), and 14% (% 95 CI; 9–21%), respectively (Table 3).

The prevalence of depression in veteran military personnel in the three countries of the United States, Croatia and Canada was calculated and the results were reported in Table 3. The results of subgroup analysis showed that the majority of studies, the prevalence of which after meta-analysis was 20% (with a confidence interval of 18 to 22%), to determine the prevalence of this outcome in veteran military personnel had been performed in the United States. The outcome prevalence in veteran military personnel in Canada and Croatia was 13 and 4%, respectively. The rest of the studies had been individually conducted in only one country and because of their number, they could not be used for meta-analysis (Table 3).

#### Publication bias, and meta-regression in studies related to veterans

The results of the publication bias were shown in Fig. 2 for studies related to veterans. The results of the Eggers test presented that bias occurred in calculating the prevalence of depression in veterans (B: 8.95, SE: 0.54, P: 0.001) (Fig. 2). In meta-regression analysis, the effect of military age on prevalence was examined and analyzed, which showed that age did not have a significant effect on the prevalence of depression in military veterans.

#### Prevalence of suicide in the military

**Fig. 3:**
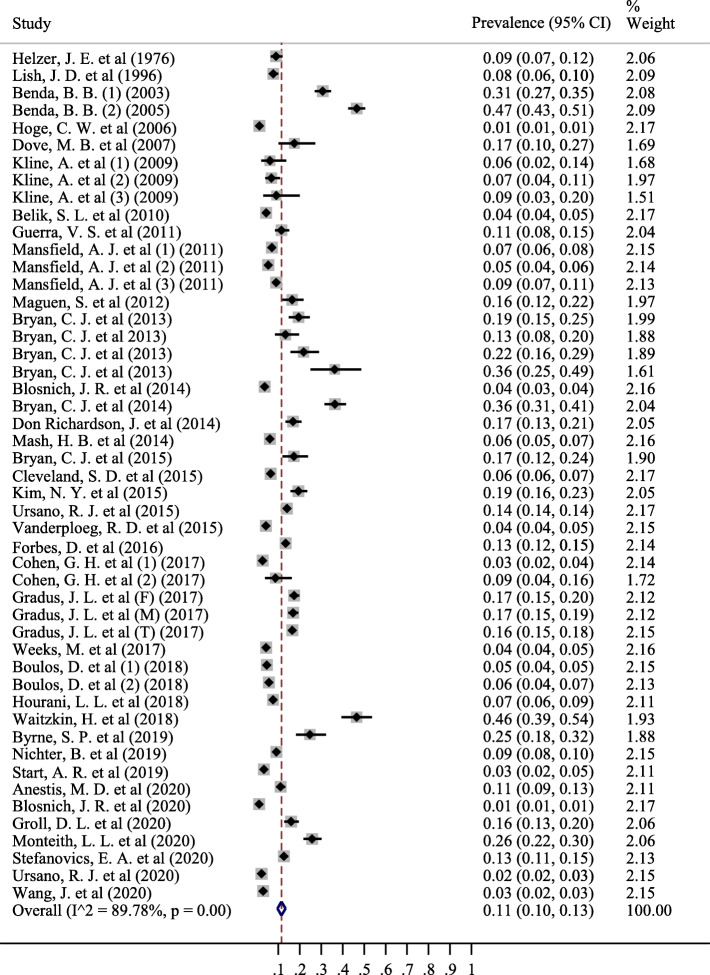
The pooled prevalence of Suicide thought in all military

**Fig. 4:**
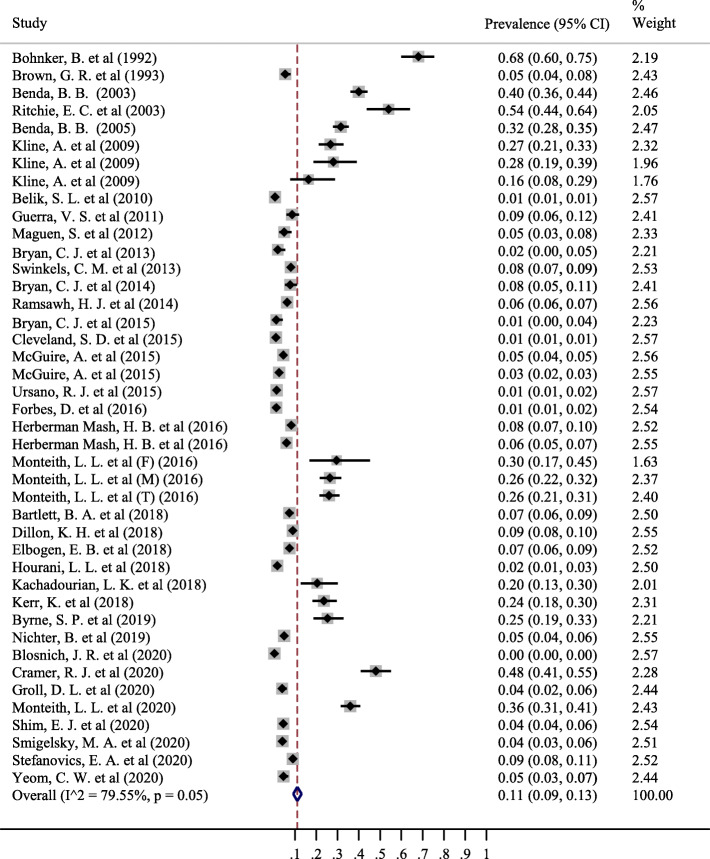
The pooled prevalence of Suicide attempted in all military

The results of this study demonstrated that 49 studies related to the prevalence of suicidal ideation in the military and 42 studies related to the prevalence of suicide attempts in the military were included in the meta-analysis. The sample size in studies related to suicidal ideation was 759,374 people, of whom a total of 20,065 individuals had suicidal ideation. However, the sample size in studies related to suicide attempts was equal to 438,890 people, of whom 5471 people had attempted suicide. The results of meta-analysis showed that the pooled prevalence of suicidal ideation in the entire military was 11% (% 95 CI; 10–13%) (Fig. 3). The pooled prevalence of suicide attempts in all military was equal to the prevalence of suicidal ideation 11% (% 95 CI; 9–13%) (Fig. 4).

To accurately estimate the prevalence of suicidal ideation in the military and to find the source of heterogeneity in the study, the subgroup analysis was performed based on whether the military person was serving or a veteran at that time, the study sampling method (random or convinces), the military service location, the statistical population of the study in terms of the presence of various diseases or being healthy, gender, and finally the tools used to measure suicide ideation and attempts. The results were shown in Table 4. As can be seen from the results, the pooled prevalence of suicidal ideation in veterans was higher than that in active military (14% vs. 10%). Suicidal ideation was also higher in women than men (Table 4). The pooled prevalence of suicidal ideation was higher in the air force (19%) than that in the navy and the army (Table 4). In the military with substance use, the prevalence of suicidal ideation was 18% (% 95 CI; 7–33%), which was higher than one in the military consuming alcohol with a prevalence of 9% (% 95 CI; 4–13%) (Table 4). In studies that had used multi-problem screening inventory (MPSI) and the self-injurious thoughts and behaviors interview (SITBI) to estimate suicidal ideation, the prevalence was 39% (% 95 CI; 36–41%), and 36% (% 95 CI; 32–41%), respectively, which was higher than those in studies that had used other tools to estimate the prevalence of suicidal ideation in the military (Table 4).

**Table 4:** The pooled estimate of prevalence of suicide in active duty and veteran military

Categories	No. of Studies (Sample Size)	Pooled Prevalence (% 95 CI)	Between studies heterogeneity assessment (%)			Between subgroups heterogeneity assessment (%)	
			*I*^*2*^	*P*_*Heterogenity*_	*Z*	Q	*P*_*Heterogenity*_
The prevalence of suicide thought in military							
Military Statue							
Active Duty	31 (424253)	10% (7–13%)	67.55%	0.402	12.55	2.24	0.130
Veteran	18 (335121)	14% (10–20%)	69.77%	0.329	9.59		
Sampling Method							
Convinces Sampling	40 (151199)	12% (10–15%)	57.23%	0.170	20.37	15.76	0.001
Random Sampling	9 (608175)	7% (6–9%)	74.31%	0.059	17.83		
Type of Forces							
Air Forces	2 (441)	19% (15–22%)	78.99%	0.041	17.36	30.05	0.001
Armed Forces	23 (434677)	8% (5–11%)	88.68%	0.025	9.57		
Marine Forces	2 (295715)	1% (1–2%)	93.86%	0.0001	10.98		
Military Forces	22 (28982)	16% (12–21%)	77.83%	0.041	13.22		
Population Healthy Forces	42 (757597)	11% (9–13%)	99.80%	0.0001	20.07	1.74	0.420
Forces with HIV/AIDS	–	–		–	–		
Forces with Alcohol Use	1 (93)	9% (4–13%)	–	–	4.45		
Forces with Substance Use	6 (1684)	18%(7–33%)	97.74%	0.0001	4.91		
Gender							
Total	44 (756218)	11% (9–13%)	88.72%	0.0001	20.10	12.30	0.001
Male	2 (1532)	14% (12–16%)	90.00%	0.0001	10.94		
Female	3 (1624)	20% (14–27%)	75.22%	0.017	28.78		
Tools							
BSSI-C Scale	5 (12775)	11% (7–16%)	67.96%	0.052	8.98	24.84	0.001
SCRENNER Scale	1 (669)	8% (6–10%)	–	–	13.54		
SCID DSM-IV Scale	16 (375640)	7% (5–10%)	69.80%	0.049	10.23		
MPSI Scale	2 (1225)	39% (36–41%)	55.21%	0.077	15.52		
PHQ Scale	15 (324540)	9% (6–13%)	53.01%	0.850	9.61		
SITBI Scale	2 (443)	36% (32–41%)	44.34%	0.501	13.35		
SBQ-R Scale	7 (5845)	16% (14–18%)	77.69%	0.053	12.25		
C-SSRS Scale	1 (38237)	14% (12–18%)	–	–	14.98		
Country							
USA	42 (707764)	12% (10–14%)	79.90%	0.001	19.65	21.35	0.001
Canada	6 (49560)	7% (6–10%)	76.92%	0.001	14.19		
The prevalence of suicide attempted in military							
Military Statue							
Active Duty	19 (98426)	8% (6–10%)	50.18%	0.497	12.14	10.13	0.001
Veteran	23 (340464)	15% (11–19%)	69.80%	0.122	12.59		
Sampling Method							
Convinces Sampling	35 (133437)	11% (9–13%)	77.78%	0.059	16.11	0.30	0.660
Random Sampling	7 (305453)	13% (7–20%)	64.26%	0.051	6.47		
Type of Forces							
Air Forces	4 (4851)	13% (1–35%)	79.99%	0.047	2.54		
Armed Forces	23 (121644)	12% (9–15%)	76.44%	0.044	14.50	1.27	0.260
Marine Forces	1 (100)	54% (44–64%)	–	–	15.54		
Military Forces	14 (312295)	8% (4–12%)	74.77%	0.034	6.82		
Population							
Healthy Forces	35 (435640)	9% (8–11%)	99.09%	0.0001	18.52	84.99	0.001
Forces with HIV/AIDS	1 (442)	5% (4–8%)	–	–	19.33		
Forces with Alcohol Use	1 (1210)	8% (7–10%)	–	–	14.59		
Forces with Substance Use	5 (1598)	30%(23–36%)	87.44%	0.0001	8.99		
Forces with HCV	–	–	–	–	–		
Gender							
Total	37 (429113)	11% (9–13%)	92.88%	0.0001	9.04	9.56	0.001
Male	2 (4533)	3% (2–4%)	95.91%	0.0001	2.49		
Female	3 (5244)	21% (1–53%)	98.49%	0.0001	10.75		
Tools							
BSSI-C Scale	6 (9800)	15% (10–22%)	66.33%	0.050	9.04	35.33	0.001
Checklist	6 (6882)	11% (5–20%)	78.31%	0.049	5.25		
SCID DSM-IV Scale	12 (373059)	5% (3–7%)	61.99%	0.041	11.15		
MPSI Scale	2 (1225)	36% (33–38%)	55.99%	0.055	43.39		
PHQ Scale	4 (4675)	9% (5–15%)	70.05%	0.039	6.54		
MINI-Plus Scale	2 (2417)	4% (4–5%)	60.44%	0.041	19.36		
SBQ-R Scale	3 (526)	11% (1–49%)	69.01%	0.050	1.42		
SITBI Scale	1 (374)	8% (5–11%)	–	–	10.00		
C-SSRS Scale	2 (38330)	1% (1–2%)	62.99%	0.034	32.48		
NR	3 (692)	22% (2–53%)	79.90%	0.045	2.76		
Country							
Australia	2 (2279)	2% (1–4%)	0.0%	0.777	12.13		
Canada	2 (37606)	1% (1–2%)	0.0%	0.832	25.28		
Korea	2 (2417)	4% (3–5%)	0.0%	0.489	19.36	19.75	0.001
United Kingdom	3 (10492)	5% (2–8%)	0.0%	0.880	6.64		
USA	33 (386096)	14% (11–16%)	60.98%	0.066	16.97		

Beck Scale for Suicidal Ideation-Current (BSSI-C), Brief self-report questionnaire (SCRENNER), SCID DSM-IV Diagnoses, Multi-Problem Screening Inventory (MPSI), The 4-item Suicidal Behaviors Questionnaire-Short Form (SBQ-SF), The Patient Health Questionnaire(PHQ), The Self-Injurious Thoughts and Behaviors Interview (SITBI), The Suicidal Behaviors Questionnaire Revised (SBQ-R), The Columbia Suicidal Severity Rating Scale (C-SSRS), the Mini International Neuropsychiatric Interview Plus (MINI-Plus)

In terms of the prevalence of suicide attempts, servicemen serving in the air force were more likely to commit suicide than ones in the army (13% vs. 12%). In the present analysis, the prevalence of suicide attempts in the navy was 54%, but this was the result of a study with a sample size of 100 people that could not be trusted and compared with the prevalence of suicide attempts in other military (Table 4).

The prevalence of suicide attempts in militaries with substance use was 30% (% 95 CI; 23–36%), which was higher than the prevalence of suicide attempts in non-drug-using military. Also, the prevalence of suicide attempts was 8% in militaries consuming alcohol (% 95 CI; 7–10%) and in militaries with AIDS / HIV, it was equal to 5% (% 95 CI; 4–8%) (Table 4). Also, suicide attempts in female soldiers was more than that in male soldiers (21% vs. 3%) (Table 4).

The prevalence of suicide attempts was also analyzed based on the tools used in the studies. The results showed that after combining studies using SCID DSM-IV diagnoses, beck scale for suicidal ideation-current (BSSI-C), multi-problem screening inventory (MPSI) and the suicidal behavior questionnaire revised (SBQ-R), the prevalence was 5% (% 95 CI; 3–7%), 15% (% 95 CI; 10–22%) 36% (% 95 CI; 33–38%), 11% (% 95 CI; 1–49%), respectively (Table 4).

The prevalence of suicidal ideation in the US military was 12% with a confidence interval of 10 to 14% while in the Canadian military, it was 7% with a confidence interval of 6 to 10%. The prevalence of suicide attempts in the US military was also higher than that in the Canadian, Australian, British and Korean military (Table 4).

#### Publication bias, and meta-regression in studies related to the spread of suicide ideation and attempts

**Fig. 5:**
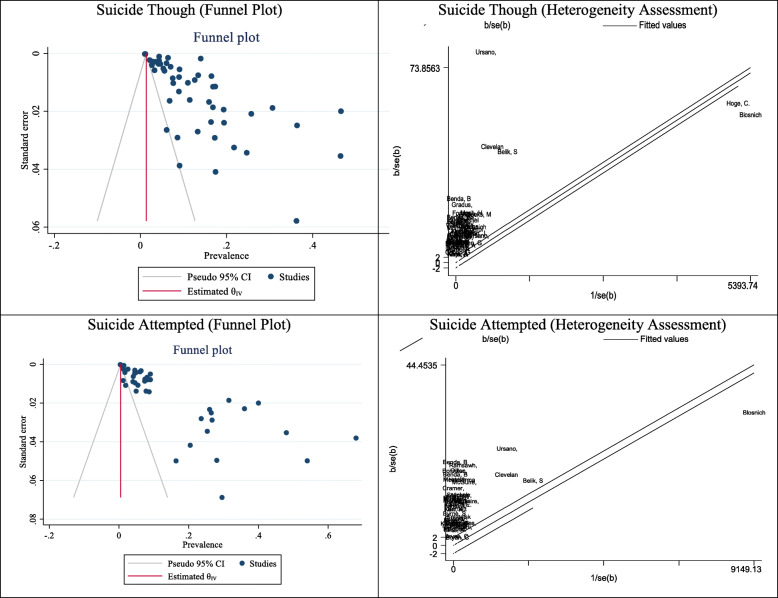
Results of Publication bias and Heterogeneity in pooled prevalence of Suicide though and attempted in all military

The results of the diffusion bias were shown in Fig. 5. The results of the Eggers test represented that diffusion bias occurred in calculating the prevalence of suicidal ideation (B: 7.59, SE: 0.99, P: 0.001) and suicide attempts (B: 7.03, SE: 0.44, P: 0.001) in the military (Fig. 5). In meta-regression analysis, the effect of military age on prevalence was examined and analyzed. The results showed that age did not have a significant effect on the prevalence of suicidal ideation and suicide attempts in the military.

## Discussion

The present study was a systematic review and meta-analysis that showed that the pooled prevalence of depression in the active military was 23%. According to the World Health Organization, the prevalence of depression in the general population is 15 to 20% [37, 38]. Therefore, it can be said that the prevalence of depression in the military community is higher than that in the general community. Feeling sad in unfavorable situations such as military situations and operational locations can be one of the reasons for the increase in the prevalence of depression or in some way the occurrence of depression and its symptoms in the military. This relationship indicates the existence of a relation between activity abnormalities, mood and thoughts with social or occupational environments [23, 39–42]. On the other hand, the military may not be very interested in their job and, therefore, they have unpleasant moods and thoughts such as sadness, grief, despair and worry, which can make a military person prone to depression [43, 44]. Military personnel often suffer from disorders in sleep, nutrition, physical exertion, concentration, as well as anorexia, and weight changes due primarily to job sensitivity and confidential activities. The presence of these behaviors and emotions over time and their stability for a long time have a negative effect on the mood of these people and can expose a military person to depression [24, 45]. In the present meta-analysis, the pooled prevalence of depression after combining studies in which the available sampling method had been used, was equal to 21% and after combining studies that had used random sampling method to collect their samples, the pooled prevalence of depression was equal to 26%. In cross-sectional studies, the sampling method should be random in order to consider samples under investigation as a good representative of the target population. In studies that had selected this type of sampling, the pooled prevalence of depression was higher. On the other hand, the results of the subgroup analysis showed that the amount of heterogeneity after the analysis based on the sampling method has decreased, which indicated that different sampling methods in meta-analysis studies were one of the sources of heterogeneity in the total pooled prevalence in the active military.

The results of the present meta-analysis represented that the prevalence of depression was higher in active servicemen in the navy than in those in the air force and the army. The navy has more professional problems in terms of special professional missions, and more psychological problems than the army and the air Force. Job-related stress, complex missions, strict rules, the possibility of injury, disability, captivity and even death are some of the issues that increase the likelihood of depression in these soldiers compared to others [46, 47]. A person’s psychological capacity includes a person’s ability to cope with the expectations and difficulties of everyday life. High psychological capacity allows a person to maintain his/her life at the desired psychological level and crystallize this ability in the form of adaptive behaviors, effective and positive actions for himself/herself. The role of psychological capacity in promoting health and well-being in all three aspects of physical, mental and social is very important. This importance becomes even more apparent when the problem becomes behavioral. In such a case, the person is not strong enough when faced with psychological pressures and obstacles in life, and as a result, his/her inappropriate behavior will be the source of all suffering and failure [48, 49]. Therefore, addressing various psychological aspects, quality of life and social relations of the military, especially the navy, in order to properly understand the conditions of these people and their families can be useful to strengthen and enhance their military capabilities and efficiency. Other reasons for the increasing prevalence of depression in the navy include family problems [50]. Over the years, research has shown that the family plays an important role in providing function and activity to individuals. Having a healthy society depends on having strong families in the society. Navy families often suffer from the stress of being away from a normal life, living in unfamiliar environments, and experiencing life outside their homelands. These may cause problems within the family, which ultimately reduce the ability of the navy and cause psychological problems such as depression [51, 52].

The stress of military jobs has major and significant consequences for the family environment. Psychological disorders between military families have been reported between 3 to 15% depending on the disorder type, while they have been reported paranoid disorders, obsessive-compulsive disorders, depression, interpersonal relationships, physical problems, and aggression, respectively [52]. According to research, it has been shown that the prevalence of these disorders in military families was higher than that in other families in the society. Factors such as workplace stress, sensitive and critical situations, high job responsibilities, job stress, unwanted relocation, problems in the family and home, lack of confidence in individual abilities, mental fatigue caused by hard work, thinking the possibility of death are some of the depression and mental distress causes in the military and their families [53, 54]. In a study entitled Environment, Lifestyle and Psychological Factors in the Health and Welfare of Military Families, the results showed that the psychological factors resulting from military missions were divided into 5 stages which included the stage before deployment, deployment, return, reinforcement and re-deployment, respectively. Military personnel and their families also experienced different psychological difficulties before, during, or after deployment to different missions. These experiences brought them many psychological norms that varied with different variables such as the location of the mission (in terms of the possibility of military conflict with hostile forces), duration of deployment, number of deployments, time between deployments, military responsibility, and the difficulty of working conditions of individuals at the time of deployment [55–57]. The same factors may lead military personnel to use drugs, and alcohol [58]. In the present meta-analysis, the prevalence of depression in the active military drug users was 37% and in the military alcohol users was 29%. Drug, and alcohol abuse can be a contributing factor to depression or other mental disorders in the military. Excessive alcohol abuse in the US military has resulted in significant financial losses. Data from 2006 showed that excessive alcohol consumption annually cost the US military 1.12 billion dollars [59, 60]. In a large survey study by Bray and Hourani, the results demonstrated that the prevalence of alcohol use in the US military was 15 to 20% [61]. Also, in terms of gender, this prevalence was different and in men, alcohol consumption was 3.5 times more than that in military women. The results of studies have shown that the prevalence of alcohol and drug use in the Navy was higher than that in the Air Force, which might be related to the high prevalence of depression in the navy [62–64]. Alcohol and substance abuse occur more frequently in war veterans. A study by Milliken and colleagues in a population-based study found that 12 to 15% of veterans experienced alcohol and substance abuse after 3 to 6 months of returning from war, which put them at risk of depression [65–67]. In the present meta-analysis, the overall pooled prevalence of depression in veterans was 20%. However, in studies that had used random sampling to collect samples, the prevalence was 22%.

The prevalence of depression was 15% in active HIV-positive servicemen and 16% in HIV-positive veterans. These military personnel, of course, suffered from depression and other mental disorders due to the existence of the disease and its difficult conditions in the society. The prevalence of depression in veterans with hepatitis C was 29%. It was noteworthy that the amount of heterogeneity during the subgroup analysis based on the healthy and unhealthy military population did not significantly decrease compared to the overall prevalence of heterogeneity, which indicated the lack of the inclusion effect of soldiers with various diseases, and healthy soldiers on the amount of heterogeneity in studies. In other words, this factor could not be a source of heterogeneity when estimating the overall prevalence of depression. However, as shown in Table 4, the type of sampling (random or available), location and place of service (the air, naval or army), and various tools for measuring the prevalence of depression were the main sources of heterogeneity when estimating general depression in the military because the amount and percentage of heterogeneity had significantly decreased when performing subgroups based on these variables.

The prevalence of suicidal ideation in the present meta-analysis in the military was equal to the prevalence of suicide attempts in the entire military. Suicidal ideation was also more common in women than in military men. According to studies conducted in the world, the prevalence of suicide and its thoughts in the military had a range from 5.8 to 28.4%, which in the present meta-analysis study was exactly equal to 11%. In the study of Farsi et al., the results showed that with increasing scores of depression, the possibility of self-harm and suicide in the military increased [68]. In the study by Hossieni et al., The prevalence of depressive disorders in military personnel who had attempted suicide was 0.7 to 1.3% [69]. The prevalence of suicidal ideation was higher in Air Force servicemen than that in Navy and Land Force servicemen. The prevalence of suicidal ideation was 18% in the military using drug, which was higher than that in the military using alcohol. Also, the prevalence of suicide attempts in drug-using military was higher than the prevalence of suicide attempts in non-drug-using military. The results of the present meta-analysis showed that the use of drugs, alcohol and diseases such as HIV and HCV could be a predisposing factor in the development of mental disorders and the development of suicidal ideation and suicide attempts in the military. In addition, there were more thoughts and attempts to commit suicide in veterans than in active and serving soldiers. One of the effective reasons for the existence of suicidal ideation and attempts in the veterans was the lack of combat and other physical activities, living at home, consuming drugs and alcohol. The results of the present meta-analysis represented that the prevalence of suicidal ideation and attempts in military personnel using drugs were equal to 18 and 30%, respectively.

Regarding the prevalence of suicidal ideation and attempts, the results of the subgroup analysis showed that the use of different tools in determining the prevalence of suicidal ideation in the military in meta-analysis studies, different sampling methods (available or random sampling), and the type of servicemen included in the study (in-service or veterans) were among the most important factors in creating heterogeneity in determining the pooled prevalence of suicidal ideation and attempts in the military after completing the entire study. The subgroup analysis was based on different countries, but most studies had been conducted in the United States. The following subgroup results showed that the prevalence of depression in the US active military was 21% (with a confidence interval of 17 to 25%) while the prevalence of depression in the Thailand and British military was higher than that in other countries, which was 39 and 30%, respectively. The prevalence of depression was higher in retired US troops than that in retired Canadian and Croatian troops. Also, the prevalence of suicidal ideation in the US military was higher than that in the Canadian, Australian, British and Korean militaries. In this analysis, the amount of heterogeneity significantly decreased in different subgroups, which indicated the role of different cultures, different military methods for training soldiers, and different military environments in various countries as the sources of heterogeneity.

In this meta-analysis, the finding of articles published from January 1990 to December 2020 was analyzed. Articles on suicide or depression in the military have been published in PubMed since 1966. But, these types of studies did not have the appropriate structure of original or cross-sectional studies (which were the main studies included in this meta-analysis). In addition, studies before 1990 did not have a suitable sample size to be able to enter the present meta-analysis. Finally, articles from 1990 to 2020 were considered to avoid creating too much heterogeneity and bias in the results. In this study, it was decided to determine the exact prevalence because meta-analysis of prevalence gives the reader and health policy makers better interpretations than the average, and this value is more tangible for health policy makers. Also, estimating the prevalence of depression and suicide can be effective and useful in estimating the burden of these diseases and in planning health programs for the military of the world.

The present meta-analysis study was the first systematic review and meta-analysis study to determine the prevalence of depressive and suicidal disorders in the entire military worldwide. Also, the exact prevalence of these disorders in the military had not been reported and this research determined the overall pooled prevalence of depression and suicidal ideation or attempts. On the other hand, the sample size in the present meta-analysis subgroup was very significant, which made the estimated prevalence in each subgroup very reliable. Other benefits of this study included determining the prevalence of depressive disorders and suicide in military personnel in various sectors, such as the navy, air, and army forces. One of the limitations of the present study was the lack of sufficient number of studies and sample sizes to determine the prevalence of depressive and suicidal disorders in servicemen with hepatitis C or other diseases. For future research, the issue of social classes, religion, and income levels need to be considered to determine the prevalence of mental disorders in the military. Also, studies on how to carry out preventive interventions, and their cost-effectiveness need to be done in order to determine effective and useful interventions in the military to prevent suicide and depression.

## Conclusion

The present study showed that the prevalence of depression and suicide (thoughts and actions) was high in the military, especially in the navy and air forces, and this prevalence was more significant. On the other hand, substance and alcohol consumption were factors that increased the prevalence of depression and ultimately led to suicide in the military. Therefore, it is necessary to develop and design training and intervention programs in order to train and increase the awareness of the military, especially veterans, in order to prevent the occurrence of suicide and mental disorders such as depression. Considering the prevalence of depression and suicide in the military consuming drugs and alcohol in the present meta-analysis study, it is necessary to implement screening and follow-up measures to identify, and prevent these two disorders (drug and alcohol consumption) in the military.

## Supplementary Information

**Additional file 1.** The search syntax in PubMed and Embase.

## Data Availability

Data is available and it can be accessed from the corresponding author with reasonable inquiry.

## Abbreviations

CI: Confidence Interval
EMBASE: Excerpta Medica dataBASE
NOS: Newcastle-Ottawa Scale
MOOSE: The Meta-Analyses of Observational Studies in Epidemiology
PRISMA: Preferred Reporting Items for Systematic Reviews and Meta-analyses
WHO: World Health Organization
DSM-IV: The diagnostic and statistical manual of mental disorders-IV
PHQ: The patient health questionnaire
SDS: The Zung self-tool report depression scale
BDI: The beck depression inventory
CES-D: The center for epidemiological studies depression
HDRS: Hamilton depression rating scale
MHI: Mental health inventory
QIDS: The quick inventory of depressive symptomatology
GDS: The geriatric depression scale
SCID DSM-IV: Structured Clinical Interview for DSM Disorders
BSSI-C: Beck scale for suicidal ideation-current
MPSI: Multi-problem screening inventory
SBQ-R: The suicidal behavior questionnaire revised
SCRENNER: Brief self-report questionnaire
MPSI: Multi-Problem Screening Inventory
SBQ-SF: The 4-item Suicidal Behaviors Questionnaire-Short Form
SITBI: The Self-Injurious Thoughts and Behaviors Interview
SBQ-R: The Suicidal Behaviors Questionnaire Revised
C-SSRS: The Columbia Suicidal Severity Rating Scale
MINI-Plus: The Mini International Neuropsychiatric Interview Plus
BSI: The Brief Symptom Inventory
HADS: The Hospital Anxiety and Depression Scale
GDS: Geriatric Depression Scale
QIDS: Quick Inventory of Depressive Symptomatology
