# Supplementary material for: The global prevalence of depression, suicide ideation, and attempts in the military forces: a systematic review and Meta-analysis of cross sectional studies
Source: BMC Psychiatry. 2021 Oct 15;21:510. doi: 10.1186/s12888-021-03526-2 (PMC8520236; doi:10.1186/s12888-021-03526-2)
Supplement: Supplementary file 1 — Additional file 1. The search syntax in PubMed and Embase. [file 12888_2021_3526_MOESM1_ESM.docx]

The search strategy in the PubMed databases

| Search Strategy in PubMed | Outcomes |
| --- | --- |
| (((((((((((((((((((((((("militaries"[Tiab] OR "military personnel"[Tiab]) OR ("military"[Tiab] AND "personnel"[Tiab])) OR "military personnel"[Tiab]) OR "military"[Tiab]) OR "military s"[Tiab]) OR (((("military personnel"[Tiab] OR ("military"[Tiab] AND "personnel"[Tiab])) OR "military personnel"[Tiab]) OR (("armed"[Tiab] AND "forces"[Tiab]) AND "personnel"[Tiab])) OR "armed forces personnel"[Tiab])) OR (((((("occupational groups"[Tiab] OR ("occupational"[Tiab] AND "groups"[Tiab])) OR "occupational groups"[Tiab]) OR "personnel"[Tiab]) OR "personnel s"[Tiab]) OR "personnel’s"[Tiab]) AND ((((("militaries"[Tiab] OR "military personnel"[Tiab]) OR ("military"[Tiab] AND "personnel"[Tiab])) OR "military personnel"[Tiab]) OR "military"[Tiab]) OR "military s"[Tiab]))) OR (((((("occupational groups"[Tiab] OR ("occupational"[Tiab] AND "groups"[Tiab])) OR "occupational groups"[Tiab]) OR "personnel"[Tiab]) OR "personnel s"[Tiab]) OR "personnel’s"[Tiab]) AND (((("military personnel"[Tiab] OR ("military"[Tiab] AND "personnel"[Tiab])) OR "military personnel"[Tiab]) OR ("armed"[Tiab] AND "forces"[Tiab])) OR "armed forces"[Tiab]))) OR (((("military personnel"[Tiab] OR ("military"[Tiab] AND "personnel"[Tiab])) OR "military personnel"[Tiab]) OR (("air"[Tiab] AND "force"[Tiab]) AND "personnel"[Tiab])) OR "air force personnel"[Tiab])) OR ((((((("force"[Tiab] OR "forceful"[Tiab]) OR "forcefulness"[Tiab]) OR "forces"[Tiab]) OR "forcing"[Tiab]) OR "forcings"[Tiab]) AND ((((("occupational groups"[Tiab] OR ("occupational"[Tiab] AND "groups"[Tiab])) OR "occupational groups"[Tiab]) OR "personnel"[Tiab]) OR "personnel s"[Tiab]) OR "personnel’s"[Tiab])) AND ("air"[Tiab] OR "air"[Tiab]))) OR (((((("occupational groups"[Tiab] OR ("occupational"[Tiab] AND "groups"[Tiab])) OR "occupational groups"[Tiab]) OR "personnel"[Tiab]) OR "personnel s"[Tiab]) OR "personnel’s"[Tiab]) AND (("air"[Tiab] OR "air"[Tiab]) AND ((((("force"[Tiab] OR "forceful"[Tiab]) OR "forcefulness"[Tiab]) OR "forces"[Tiab]) OR "forcing"[Tiab]) OR "forcings"[Tiab])))) OR (((("military personnel"[Tiab] OR ("military"[Tiab] AND "personnel"[Tiab])) OR "military personnel"[Tiab]) OR ("Army"[Tiab] AND "personnel"[Tiab])) OR "army personnel"[Tiab])) OR (((((("occupational groups"[Tiab] OR ("occupational"[Tiab] AND "groups"[Tiab])) OR "occupational groups"[Tiab]) OR "personnel"[Tiab]) OR "personnel s"[Tiab]) OR "personnel’s"[Tiab]) AND "Army"[Tiab])) OR (((((("military personnel"[Tiab] OR ("military"[Tiab] AND "personnel"[Tiab])) OR "military personnel"[Tiab]) OR "submariner"[Tiab]) OR "submariners"[Tiab]) OR "submarine"[Tiab]) OR "submarines"[Tiab])) OR (((("military personnel"[Tiab] OR ("military"[Tiab] AND "personnel"[Tiab])) OR "military personnel"[Tiab]) OR "marine"[Tiab]) OR "marines"[Tiab])) OR (((("military personnel"[Tiab] OR ("military"[Tiab] AND "personnel"[Tiab])) OR "military personnel"[Tiab]) OR ("Navy"[Tiab] AND "personnel"[Tiab])) OR "navy personnel"[Tiab])) OR (((((("occupational groups"[Tiab] OR ("occupational"[Tiab] AND "groups"[Tiab])) OR "occupational groups"[Tiab]) OR "personnel"[Tiab]) OR "personnel s"[Tiab]) OR "personnel’s"[Tiab]) AND "Navy"[Tiab])) OR ((((("military personnel"[Tiab] OR ("military"[Tiab] AND "personnel"[Tiab])) OR "military personnel"[Tiab]) OR "sailor"[Tiab]) OR "sailors"[Tiab]) OR "sailor s"[Tiab])) OR ((((("military personnel"[Tiab] OR ("military"[Tiab] AND "personnel"[Tiab])) OR "military personnel"[Tiab]) OR "sailor"[Tiab]) OR "sailors"[Tiab]) OR "sailor s"[Tiab])) OR ((((("military personnel"[Tiab] OR ("military"[Tiab] AND "personnel"[Tiab])) OR "military personnel"[Tiab]) OR "soldier"[Tiab]) OR "soldiers"[Tiab]) OR "soldier s"[Tiab])) OR ((((("military personnel"[Tiab] OR ("military"[Tiab] AND "personnel"[Tiab])) OR "military personnel"[Tiab]) OR "soldier"[Tiab]) OR "soldiers"[Tiab]) OR "soldier s"[Tiab])) OR (((("military personnel"[Tiab] OR ("military"[Tiab] AND "personnel"[Tiab])) OR "military personnel"[Tiab]) OR ("military"[Tiab] AND "deployment"[Tiab])) OR "military deployment"[Tiab])) OR ((((("deployability"[Tiab] OR "deployable"[Tiab]) OR "deployers"[Tiab]) OR "deployment"[Tiab]) OR "deployments"[Tiab]) AND ((((("militaries"[Tiab] OR "military personnel"[Tiab]) OR ("military"[Tiab] AND "personnel"[Tiab])) OR "military personnel"[Tiab]) OR "military"[Tiab]) OR "military s"[Tiab]))) OR (((("military personnel"[Tiab] OR ("military"[Tiab] AND "personnel"[Tiab])) OR "military personnel"[Tiab]) OR ("coast"[Tiab] AND "guard"[Tiab])) OR "coast guard"[Tiab])) AND ((((((((((((((((("suicid"[Tiab] OR "suicidal"[Tiab]) OR "suicidality"[Tiab]) OR "suicidally"[Tiab]) OR "suicidals"[Tiab]) OR "suicide"[Tiab]) OR "suicide"[Tiab]) OR "suicides"[Tiab]) OR "suicide s"[Tiab]) OR "suicided"[Tiab]) OR "suiciders"[Tiab]) OR (((((((((("suicid"[Tiab] OR "suicidal"[Tiab]) OR "suicidality"[Tiab]) OR "suicidally"[Tiab]) OR "suicidals"[Tiab]) OR "suicide"[Tiab]) OR "suicide"[Tiab]) OR "suicides"[Tiab]) OR "suicide s"[Tiab]) OR "suicided"[Tiab]) OR "suiciders"[Tiab])) OR ((("suicide, attempted"[Tiab] OR ("suicide"[Tiab] AND "attempted"[Tiab])) OR "attempted suicide"[Tiab]) OR ("attempted"[Tiab] AND "suicide"[Tiab]))) OR (((((("parasuicidal"[Tiab] OR "parasuicidality"[Tiab]) OR "suicide, attempted"[Tiab]) OR ("suicide"[Tiab] AND "attempted"[Tiab])) OR "attempted suicide"[Tiab]) OR "parasuicide"[Tiab]) OR "parasuicides"[Tiab])) OR (((((("parasuicidal"[Tiab] OR "parasuicidality"[Tiab]) OR "suicide, attempted"[Tiab]) OR ("suicide"[Tiab] AND "attempted"[Tiab])) OR "attempted suicide"[Tiab]) OR "parasuicide"[Tiab]) OR "parasuicides"[Tiab])) OR (((("suicide, completed"[Tiab] OR ("suicide"[Tiab] AND "completed"[Tiab])) OR "completed suicide"[Tiab]) OR ("completed"[Tiab] AND "suicides"[Tiab])) OR "completed suicides"[Tiab])) OR ((((((((((("suicid"[Tiab] OR "suicidal"[Tiab]) OR "suicidality"[Tiab]) OR "suicidally"[Tiab]) OR "suicidals"[Tiab]) OR "suicide"[Tiab]) OR "suicide"[Tiab]) OR "suicides"[Tiab]) OR "suicide s"[Tiab]) OR "suicided"[Tiab]) OR "suiciders"[Tiab]) AND ((((((((("complete"[Tiab] OR "completed"[Tiab]) OR "completely"[Tiab]) OR "completeness"[Tiab]) OR "completer"[Tiab]) OR "completers"[Tiab]) OR "completes"[Tiab]) OR "completing"[Tiab]) OR "completion"[Tiab]) OR "completions"[Tiab]))) OR ((("suicide, completed"[Tiab] OR ("suicide"[Tiab] AND "completed"[Tiab])) OR "completed suicide"[Tiab]) OR ("completed"[Tiab] AND "suicide"[Tiab]))) | Suicide |
| (((((((((((((((((((((((("militaries"[Tiab] OR "military personnel"[Tiab]) OR ("military"[Tiab] AND "personnel"[Tiab])) OR "military personnel"[Tiab]) OR "military"[Tiab]) OR "military s"[Tiab]) OR (((("military personnel"[Tiab] OR ("military"[Tiab] AND "personnel"[Tiab])) OR "military personnel"[Tiab]) OR (("armed"[Tiab] AND "forces"[Tiab]) AND "personnel"[Tiab])) OR "armed forces personnel"[Tiab])) OR (((((("occupational groups"[Tiab] OR ("occupational"[Tiab] AND "groups"[Tiab])) OR "occupational groups"[Tiab]) OR "personnel"[Tiab]) OR "personnel s"[Tiab]) OR "personnels"[Tiab]) AND ((((("militaries"[Tiab] OR "military personnel"[Tiab]) OR ("military"[Tiab] AND "personnel"[Tiab])) OR "military personnel"[Tiab]) OR "military"[Tiab]) OR "military s"[Tiab]))) OR (((((("occupational groups"[Tiab] OR ("occupational"[Tiab] AND "groups"[Tiab])) OR "occupational groups"[Tiab]) OR "personnel"[Tiab]) OR "personnel s"[Tiab]) OR "personnels"[Tiab]) AND (((("military personnel"[Tiab] OR ("military"[Tiab] AND "personnel"[Tiab])) OR "military personnel"[Tiab]) OR ("armed"[Tiab] AND "forces"[Tiab])) OR "armed forces"[Tiab]))) OR (((("military personnel"[Tiab] OR ("military"[Tiab] AND "personnel"[Tiab])) OR "military personnel"[Tiab]) OR (("air"[Tiab] AND "force"[Tiab]) AND "personnel"[Tiab])) OR "air force personnel"[Tiab])) OR ((((((("force"[Tiab] OR "forceful"[Tiab]) OR "forcefulness"[Tiab]) OR "forces"[Tiab]) OR "forcing"[Tiab]) OR "forcings"[Tiab]) AND ((((("occupational groups"[Tiab] OR ("occupational"[Tiab] AND "groups"[Tiab])) OR "occupational groups"[Tiab]) OR "personnel"[Tiab]) OR "personnel s"[Tiab]) OR "personnels"[Tiab])) AND ("air"[Tiab] OR "air"[Tiab]))) OR (((((("occupational groups"[Tiab] OR ("occupational"[Tiab] AND "groups"[Tiab])) OR "occupational groups"[Tiab]) OR "personnel"[Tiab]) OR "personnel s"[Tiab]) OR "personnels"[Tiab]) AND (("air"[Tiab] OR "air"[Tiab]) AND ((((("force"[Tiab] OR "forceful"[Tiab]) OR "forcefulness"[Tiab]) OR "forces"[Tiab]) OR "forcing"[Tiab]) OR "forcings"[Tiab])))) OR (((("military personnel"[Tiab] OR ("military"[Tiab] AND "personnel"[Tiab])) OR "military personnel"[Tiab]) OR ("Army"[Tiab] AND "personnel"[Tiab])) OR "army personnel"[Tiab])) OR (((((("occupational groups"[Tiab] OR ("occupational"[Tiab] AND "groups"[Tiab])) OR "occupational groups"[Tiab]) OR "personnel"[Tiab]) OR "personnel s"[Tiab]) OR "personnels"[Tiab]) AND "Army"[Tiab])) OR (((((("military personnel"[Tiab] OR ("military"[Tiab] AND "personnel"[Tiab])) OR "military personnel"[Tiab]) OR "submariner"[Tiab]) OR "submariners"[Tiab]) OR "submarine"[Tiab]) OR "submarines"[Tiab])) OR (((("military personnel"[Tiab] OR ("military"[Tiab] AND "personnel"[Tiab])) OR "military personnel"[Tiab]) OR "marine"[Tiab]) OR "marines"[Tiab])) OR (((("military personnel"[Tiab] OR ("military"[Tiab] AND "personnel"[Tiab])) OR "military personnel"[Tiab]) OR ("Navy"[Tiab] AND "personnel"[Tiab])) OR "navy personnel"[Tiab])) OR (((((("occupational groups"[Tiab] OR ("occupational"[Tiab] AND "groups"[Tiab])) OR "occupational groups"[Tiab]) OR "personnel"[Tiab]) OR "personnel s"[Tiab]) OR "personnels"[Tiab]) AND "Navy"[Tiab])) OR ((((("military personnel"[Tiab] OR ("military"[Tiab] AND "personnel"[Tiab])) OR "military personnel"[Tiab]) OR "sailor"[Tiab]) OR "sailors"[Tiab]) OR "sailor s"[Tiab])) OR ((((("military personnel"[Tiab] OR ("military"[Tiab] AND "personnel"[Tiab])) OR "military personnel"[Tiab]) OR "sailor"[Tiab]) OR "sailors"[Tiab]) OR "sailor s"[Tiab])) OR ((((("military personnel"[Tiab] OR ("military"[Tiab] AND "personnel"[Tiab])) OR "military personnel"[Tiab]) OR "soldier"[Tiab]) OR "soldiers"[Tiab]) OR "soldier s"[Tiab])) OR ((((("military personnel"[Tiab] OR ("military"[Tiab] AND "personnel"[Tiab])) OR "military personnel"[Tiab]) OR "soldier"[Tiab]) OR "soldiers"[Tiab]) OR "soldier s"[Tiab])) OR (((("military personnel"[Tiab] OR ("military"[Tiab] AND "personnel"[Tiab])) OR "military personnel"[Tiab]) OR ("military"[Tiab] AND "deployment"[Tiab])) OR "military deployment"[Tiab])) OR ((((("deployability"[Tiab] OR "deployable"[Tiab]) OR "deployers"[Tiab]) OR "deployment"[Tiab]) OR "deployments"[Tiab]) AND ((((("militaries"[Tiab] OR "military personnel"[Tiab]) OR ("military"[Tiab] AND "personnel"[Tiab])) OR "military personnel"[Tiab]) OR "military"[Tiab]) OR "military s"[Tiab]))) OR (((("military personnel"[Tiab] OR ("military"[Tiab] AND "personnel"[Tiab])) OR "military personnel"[Tiab]) OR ("coast"[Tiab] AND "guard"[Tiab])) OR "coast guard"[Tiab])) AND ((((((((((((((((((((((((((((((((((((((((((("depressed"[Tiab] OR "depression"[Tiab]) OR "depression"[Tiab]) OR "depressions"[Tiab]) OR "depression s"[Tiab]) OR "depressive disorder"[Tiab]) OR ("depressive"[Tiab] AND "disorder"[Tiab])) OR "depressive disorder"[Tiab]) OR "depressivity"[Tiab]) OR "depressive"[Tiab]) OR "depressively"[Tiab]) OR "depressiveness"[Tiab]) OR "depressives"[Tiab]) OR ((("depression"[Tiab] OR "depression"[Tiab]) OR ("depressive"[Tiab] AND "symptoms"[Tiab])) OR "depressive symptoms"[Tiab])) OR ((("depression"[Tiab] OR "depression"[Tiab]) OR ("depressive"[Tiab] AND "symptom"[Tiab])) OR "depressive symptom"[Tiab])) OR ((((((("diagnosis"[MeSH Subheading] OR "diagnosis"[Tiab]) OR "symptoms"[Tiab]) OR "diagnosis"[Tiab]) OR "symptom"[Tiab]) OR "symptom s"[Tiab]) OR "symptomes"[Tiab]) AND (((((((((((("depressed"[Tiab] OR "depression"[Tiab]) OR "depression"[Tiab]) OR "depressions"[Tiab]) OR "depression s"[Tiab]) OR "depressive disorder"[Tiab]) OR ("depressive"[Tiab] AND "disorder"[Tiab])) OR "depressive disorder"[Tiab]) OR "depressivity"[Tiab]) OR "depressive"[Tiab]) OR "depressively"[Tiab]) OR "depressiveness"[Tiab]) OR "depressives"[Tiab]))) OR ((((((("diagnosis"[MeSH Subheading] OR "diagnosis"[Tiab]) OR "symptoms"[Tiab]) OR "diagnosis"[Tiab]) OR "symptom"[Tiab]) OR "symptom s"[Tiab]) OR "symptomes"[Tiab]) AND (((((((((((("depressed"[Tiab] OR "depression"[Tiab]) OR "depression"[Tiab]) OR "depressions"[Tiab]) OR "depression s"[Tiab]) OR "depressive disorder"[Tiab]) OR ("depressive"[Tiab] AND "disorder"[Tiab])) OR "depressive disorder"[Tiab]) OR "depressivity"[Tiab]) OR "depressive"[Tiab]) OR "depressively"[Tiab]) OR "depressiveness"[Tiab]) OR "depressives"[Tiab]))) OR ((("depression"[Tiab] OR "depression"[Tiab]) OR ("emotional"[Tiab] AND "depression"[Tiab])) OR "emotional depression"[Tiab])) OR ((((((((((((("depressed"[Tiab] OR "depression"[Tiab]) OR "depression"[Tiab]) OR "depressions"[Tiab]) OR "depression s"[Tiab]) OR "depressive disorder"[Tiab]) OR ("depressive"[Tiab] AND "disorder"[Tiab])) OR "depressive disorder"[Tiab]) OR "depressivity"[Tiab]) OR "depressive"[Tiab]) OR "depressively"[Tiab]) OR "depressiveness"[Tiab]) OR "depressives"[Tiab]) AND (((((("emoting"[Tiab] OR "emotion s"[Tiab]) OR "emotions"[Tiab]) OR "emotions"[Tiab]) OR "emotion"[Tiab]) OR "emotional"[Tiab]) OR "emotive"[Tiab]))) OR ((((((((((((("depressed"[Tiab] OR "depression"[Tiab]) OR "depression"[Tiab]) OR "depressions"[Tiab]) OR "depression s"[Tiab]) OR "depressive disorder"[Tiab]) OR ("depressive"[Tiab] AND "disorder"[Tiab])) OR "depressive disorder"[Tiab]) OR "depressivity"[Tiab]) OR "depressive"[Tiab]) OR "depressively"[Tiab]) OR "depressiveness"[Tiab]) OR "depressives"[Tiab]) AND (((((("emoting"[Tiab] OR "emotion s"[Tiab]) OR "emotions"[Tiab]) OR "emotions"[Tiab]) OR "emotion"[Tiab]) OR "emotional"[Tiab]) OR "emotive"[Tiab]))) OR (("depression"[Tiab] OR "depression"[Tiab]) OR ("emotional"[Tiab] AND "depressions"[Tiab]))) OR (((("depressive disorder"[Tiab] OR ("depressive"[Tiab] AND "disorder"[Tiab])) OR "depressive disorder"[Tiab]) OR ("depressive"[Tiab] AND "disorders"[Tiab])) OR "depressive disorders"[Tiab])) OR (((("depressive disorder"[Tiab] OR ("depressive"[Tiab] AND "disorder"[Tiab])) OR "depressive disorder"[Tiab]) OR ("unipolar"[Tiab] AND "depressions"[Tiab])) OR "unipolar depressions"[Tiab])) OR ((((((((((((("depressed"[Tiab] OR "depression"[Tiab]) OR "depression"[Tiab]) OR "depressions"[Tiab]) OR "depression s"[Tiab]) OR "depressive disorder"[Tiab]) OR ("depressive"[Tiab] AND "disorder"[Tiab])) OR "depressive disorder"[Tiab]) OR "depressivity"[Tiab]) OR "depressive"[Tiab]) OR "depressively"[Tiab]) OR "depressiveness"[Tiab]) OR "depressives"[Tiab]) AND ("unipolar"[Tiab] OR "unipolars"[Tiab]))) OR (((("depressive disorder"[Tiab] OR ("depressive"[Tiab] AND "disorder"[Tiab])) OR "depressive disorder"[Tiab]) OR ("unipolar"[Tiab] AND "depression"[Tiab])) OR "unipolar depression"[Tiab])) OR (((("depressive disorder"[Tiab] OR ("depressive"[Tiab] AND "disorder"[Tiab])) OR "depressive disorder"[Tiab]) OR "melancholia"[Tiab]) OR "melancholias"[Tiab])) OR (((("depressive disorder"[Tiab] OR ("depressive"[Tiab] AND "disorder"[Tiab])) OR "depressive disorder"[Tiab]) OR "melancholia"[Tiab]) OR "melancholias"[Tiab])) OR (((("depressive disorder"[Tiab] OR ("depressive"[Tiab] AND "disorder"[Tiab])) OR "depressive disorder"[Tiab]) OR ("neurotic"[Tiab] AND "depressions"[Tiab])) OR "neurotic depressions"[Tiab])) OR (((("depressive disorder"[Tiab] OR ("depressive"[Tiab] AND "disorder"[Tiab])) OR "depressive disorder"[Tiab]) OR ("neurotic"[Tiab] AND "depression"[Tiab])) OR "neurotic depression"[Tiab])) OR ((((((((((((("depressed"[Tiab] OR "depression"[Tiab]) OR "depression"[Tiab]) OR "depressions"[Tiab]) OR "depression s"[Tiab]) OR "depressive disorder"[Tiab]) OR ("depressive"[Tiab] AND "disorder"[Tiab])) OR "depressive disorder"[Tiab]) OR "depressivity"[Tiab]) OR "depressive"[Tiab]) OR "depressively"[Tiab]) OR "depressiveness"[Tiab]) OR "depressives"[Tiab]) AND ((("neurotic"[Tiab] OR "neurotical"[Tiab]) OR "neurotically"[Tiab]) OR "neurotics"[Tiab]))) OR ((((((((((((("depressed"[Tiab] OR "depression"[Tiab]) OR "depression"[Tiab]) OR "depressions"[Tiab]) OR "depression s"[Tiab]) OR "depressive disorder"[Tiab]) OR ("depressive"[Tiab] AND "disorder"[Tiab])) OR "depressive disorder"[Tiab]) OR "depressivity"[Tiab]) OR "depressive"[Tiab]) OR "depressively"[Tiab]) OR "depressiveness"[Tiab]) OR "depressives"[Tiab]) AND ((("neurotic"[Tiab] OR "neurotical"[Tiab]) OR "neurotically"[Tiab]) OR "neurotics"[Tiab]))) OR ((((((((("syndrom"[Tiab] OR "syndromal"[Tiab]) OR "syndromally"[Tiab]) OR "syndrome"[Tiab]) OR "syndrome"[Tiab]) OR "syndromes"[Tiab]) OR "syndrome s"[Tiab]) OR "syndromic"[Tiab]) OR "syndroms"[Tiab]) AND (((((((((((("depressed"[Tiab] OR "depression"[Tiab]) OR "depression"[Tiab]) OR "depressions"[Tiab]) OR "depression s"[Tiab]) OR "depressive disorder"[Tiab]) OR ("depressive"[Tiab] AND "disorder"[Tiab])) OR "depressive disorder"[Tiab]) OR "depressivity"[Tiab]) OR "depressive"[Tiab]) OR "depressively"[Tiab]) OR "depressiveness"[Tiab]) OR "depressives"[Tiab]))) OR ((((((((("syndrom"[Tiab] OR "syndromal"[Tiab]) OR "syndromally"[Tiab]) OR "syndrome"[Tiab]) OR "syndrome"[Tiab]) OR "syndromes"[Tiab]) OR "syndrome s"[Tiab]) OR "syndromic"[Tiab]) OR "syndroms"[Tiab]) AND (((((((((((("depressed"[Tiab] OR "depression"[Tiab]) OR "depression"[Tiab]) OR "depressions"[Tiab]) OR "depression s"[Tiab]) OR "depressive disorder"[Tiab]) OR ("depressive"[Tiab] AND "disorder"[Tiab])) OR "depressive disorder"[Tiab]) OR "depressivity"[Tiab]) OR "depressive"[Tiab]) OR "depressively"[Tiab]) OR "depressiveness"[Tiab]) OR "depressives"[Tiab]))) OR (((("depressive disorder"[Tiab] OR ("depressive"[Tiab] AND "disorder"[Tiab])) OR "depressive disorder"[Tiab]) OR ("depressive"[Tiab] AND "syndromes"[Tiab])) OR "depressive syndromes"[Tiab])) OR (((("depressive disorder"[Tiab] OR ("depressive"[Tiab] AND "disorder"[Tiab])) OR "depressive disorder"[Tiab]) OR ("depressive"[Tiab] AND "syndrome"[Tiab])) OR "depressive syndrome"[Tiab])) OR (((("depressive disorder"[Tiab] OR ("depressive"[Tiab] AND "disorder"[Tiab])) OR "depressive disorder"[Tiab]) OR ("endogenous"[Tiab] AND "depressions"[Tiab])) OR "endogenous depressions"[Tiab])) OR (((("depressive disorder"[Tiab] OR ("depressive"[Tiab] AND "disorder"[Tiab])) OR "depressive disorder"[Tiab]) OR ("endogenous"[Tiab] AND "depression"[Tiab])) OR "endogenous depression"[Tiab])) OR ((((((((((((("depressed"[Tiab] OR "depression"[Tiab]) OR "depression"[Tiab]) OR "depressions"[Tiab]) OR "depression s"[Tiab]) OR "depressive disorder"[Tiab]) OR ("depressive"[Tiab] AND "disorder"[Tiab])) OR "depressive disorder"[Tiab]) OR "depressivity"[Tiab]) OR "depressive"[Tiab]) OR "depressively"[Tiab]) OR "depressiveness"[Tiab]) OR "depressives"[Tiab]) AND ((((((("endogen"[Tiab] OR "endogene"[Tiab]) OR "endogeneous"[Tiab]) OR "endogeneously"[Tiab]) OR "endogenes"[Tiab]) OR "endogenic"[Tiab]) OR "endogenous"[Tiab]) OR "endogenously"[Tiab]))) OR ((((((((((((("depressed"[Tiab] OR "depression"[Tiab]) OR "depression"[Tiab]) OR "depressions"[Tiab]) OR "depression s"[Tiab]) OR "depressive disorder"[Tiab]) OR ("depressive"[Tiab] AND "disorder"[Tiab])) OR "depressive disorder"[Tiab]) OR "depressivity"[Tiab]) OR "depressive"[Tiab]) OR "depressively"[Tiab]) OR "depressiveness"[Tiab]) OR "depressives"[Tiab]) AND ((((((("endogen"[Tiab] OR "endogene"[Tiab]) OR "endogeneous"[Tiab]) OR "endogeneously"[Tiab]) OR "endogenes"[Tiab]) OR "endogenic"[Tiab]) OR "endogenous"[Tiab]) OR "endogenously"[Tiab]))) OR (((("neurotic disorders"[Tiab] OR ("neurotic"[Tiab] AND "disorders"[Tiab])) OR "neurotic disorders"[Tiab]) OR "neuroses"[Tiab]) AND (((((((((((("depressed"[Tiab] OR "depression"[Tiab]) OR "depression"[Tiab]) OR "depressions"[Tiab]) OR "depression s"[Tiab]) OR "depressive disorder"[Tiab]) OR ("depressive"[Tiab] AND "disorder"[Tiab])) OR "depressive disorder"[Tiab]) OR "depressivity"[Tiab]) OR "depressive"[Tiab]) OR "depressively"[Tiab]) OR "depressiveness"[Tiab]) OR "depressives"[Tiab]))) OR (((("depressive disorder"[Tiab] OR ("depressive"[Tiab] AND "disorder"[Tiab])) OR "depressive disorder"[Tiab]) OR ("depressive"[Tiab] AND "neurosis"[Tiab])) OR "depressive neurosis"[Tiab])) OR (((("depressive disorder"[Tiab] OR ("depressive"[Tiab] AND "disorder"[Tiab])) OR "depressive disorder"[Tiab]) OR ("depressive"[Tiab] AND "neuroses"[Tiab])) OR "depressive neuroses"[Tiab])) OR (((("neurotic disorders"[Tiab] OR ("neurotic"[Tiab] AND "disorders"[Tiab])) OR "neurotic disorders"[Tiab]) OR "neurosis"[Tiab]) AND (((((((((((("depressed"[Tiab] OR "depression"[Tiab]) OR "depression"[Tiab]) OR "depressions"[Tiab]) OR "depression s"[Tiab]) OR "depressive disorder"[Tiab]) OR ("depressive"[Tiab] AND "disorder"[Tiab])) OR "depressive disorder"[Tiab]) OR "depressivity"[Tiab]) OR "depressive"[Tiab]) OR "depressively"[Tiab]) OR "depressiveness"[Tiab]) OR "depressives"[Tiab]))) OR (((((("disease"[Tiab] OR "disease"[Tiab]) OR "disorder"[Tiab]) OR "disorders"[Tiab]) OR "disorder s"[Tiab]) OR "disordes"[Tiab]) AND (((((((((((("depressed"[Tiab] OR "depression"[Tiab]) OR "depression"[Tiab]) OR "depressions"[Tiab]) OR "depression s"[Tiab]) OR "depressive disorder"[Tiab]) OR ("depressive"[Tiab] AND "disorder"[Tiab])) OR "depressive disorder"[Tiab]) OR "depressivity"[Tiab]) OR "depressive"[Tiab]) OR "depressively"[Tiab]) OR "depressiveness"[Tiab]) OR "depressives"[Tiab]))) | Depression |

**Search Strategy (Embase)**

| **Outcomes** | **Search Strategy** |
| --- | --- |
| **Depression** | ('military personnel'/exp OR 'active duty personnel' OR 'air force personnel' OR 'air force recruits' OR 'air force staff' OR 'airforce recruits' OR 'armed forces personnel' OR 'armed forces staff' OR 'army personnel' OR 'army recruits' OR 'army staff' OR 'enlisted personnel' OR 'infantry recruits' OR 'infantryman' OR 'military people' OR 'military personnel' OR 'military recruit' OR 'military selection' OR 'military service member' OR 'military service members' OR 'military service personnel' OR 'military service persons' OR 'military service women' OR 'military serviceman' OR 'military servicemember' OR 'military servicemembers' OR 'military servicemen' OR 'military servicepersons' OR 'military servicewomen' OR 'military staff' OR 'military trainee' OR 'military trainees' OR 'navy personnel' OR 'navy recruits' OR 'navy staff' OR 'reserve personnel' OR 'soldier' OR 'special forces personnel') AND ('depression'/exp OR 'central depression' OR 'clinical depression' OR 'depression' OR 'depressive disease' OR 'depressive disorder' OR 'depressive episode' OR 'depressive illness' OR 'depressive personality disorder' OR 'depressive state' OR 'depressive symptom' OR 'depressive syndrome' OR 'mental depression' OR 'parental depression') |
| **Suicide** | ('military personnel'/exp OR 'active duty personnel' OR 'air force personnel' OR 'air force recruits' OR 'air force staff' OR 'airforce recruits' OR 'armed forces personnel' OR 'armed forces staff' OR 'army personnel' OR 'army recruits' OR 'army staff' OR 'enlisted personnel' OR 'infantry recruits' OR 'infantryman' OR 'military people' OR 'military personnel' OR 'military recruit' OR 'military selection' OR 'military service member' OR 'military service members' OR 'military service personnel' OR 'military service persons' OR 'military service women' OR 'military serviceman' OR 'military servicemember' OR 'military servicemembers' OR 'military servicemen' OR 'military servicepersons' OR 'military servicewomen' OR 'military staff' OR 'military trainee' OR 'military trainees' OR 'navy personnel' OR 'navy recruits' OR 'navy staff' OR 'reserve personnel' OR 'soldier' OR 'special forces personnel') AND ('suicide attempt'/exp OR 'attempted suicide' OR 'parasuicide' OR 'suicidal attempt' OR 'suicide attempt' OR 'suicide, attempted' OR 'tentamen suicidi' OR 'suicidal behavior'/exp OR 'behavior, suicidal' OR 'behaviour, suicidal' OR 'suicidal behavior' OR 'suicidal behaviour' OR 'suicidality' OR 'suicide'/exp OR 'self-killing' OR 'suicidal poisoning' OR 'suicide' OR 'suicide, completed') |
